# Signature construction and molecular subtype identification based on immune-related genes for better prediction of prognosis in hepatocellular carcinoma

**DOI:** 10.1186/s12920-023-01558-z

**Published:** 2023-06-14

**Authors:** Liang Sun, Zhengyi Wu, Cairong Dong, Shian Yu, He Huang, Zhendong Chen, Zhipeng Wu, Xiangbao Yin

**Affiliations:** Department of Hepatobiliary Surgery, the Second Affiliated Hospital of Nanchang University, Nanchang, China

**Keywords:** Hepatocellular carcinoma, Prognostic model, Bioinformatics, Immune microenvironment, Immunotherapy

## Abstract

**Objective:**

Hepatocellular carcinoma (HCC) immunotherapy is a focus of current research. We established a model that can effectively predict the prognosis and efficacy of HCC immunotherapy by analyzing the immune genes of HCC.

**Methods:**

Through the data mining of hepatocellular carcinoma in The Cancer Genome Atlas (TCGA), the immune genes with differences in tumor and normal tissues are screened, and then the univariate regression analysis is carried out to screen the immune genes with differences related to prognosis. The prognosis model of immune related genes is constructed by using the minimum absolute contraction and selection operator (lasso) Cox regression model in the TCGA training set data, The risk score of each sample was calculated, and the survival was compared with the Kaplan Meier curve and the receiver operating characteristic (ROC) curve to evaluate the predictive ability. Data sets from ICGC and TCGA were used to verify the reliability of signatures. The correlation between clinicopathological features, immune infiltration, immune escape and risk score was analyzed.

**Results:**

Seven immune genes were finally determined as the prognostic model of liver cancer. According to these 7 genes, the samples were divided into the high and low risk groups, and the results suggested that the high-risk group had a poorer prognosis, lower risk of immune escape, and better immunotherapy effect. In addition, the expression of TP53 and MSI was positively correlated in the high-risk group. Consensus clustering was performed to identify two main molecular subtypes (named clusters 1 and 2) based on the signature. It was found that compared with cluster 1, better survival outcome was observed in cluster 2.

**Conclusion:**

Signature construction and molecular subtype identification of immune-related genes could be used to predict the prognosis of HCC, which may provide a specific reference for the development of novel biomarkers for HCC immunotherapy.

**Supplementary Information:**

The online version contains supplementary material available at 10.1186/s12920-023-01558-z.

## Introduction

Hepatocellular carcinoma (HCC) is currently the sixth most common tumor in the world and the fourth most common in terms of mortality [1]. HCC is expected to become the third leading cause of cancer-related deaths by 2030, according to epidemiology [2]. Despite known risk factors for HCC, including hepatitis B, alcoholism and cirrhosis, the incidence of HCC remains high worldwide. At present, there are many ways to treat hepatocellular carcinoma, among which surgical resection is the main method, and intervention, targeted drugs and immunotherapy are also important treatment methods. However, the therapeutic effect of hepatocellular carcinoma is still poor, and its recurrence rate and mortality rate have not been effectively controlled [3]. Only 30% of HCC patients are reported to be in the early stages suitable for radical surgery. In addition, the efficacy of chemotherapeutic agents and targeted agents for advanced HCC is still limited [4].

Although currently targeted drug therapy for unresectable HCC has been proven to be effective [5]. Such as sorafenib, lovastinib and the vascular endothelial growth factor (VEGF) inhibitor ramuzumab are widely used in clinical applications [6–8]. However, we found that all of these drugs had drug reactions of varying degrees, such as skin itching, gastrointestinal reactions, and elevated blood pressure [9]. In addition, long-term use of the body is easy to produce drug resistance, its treatment effect is not as expected [10]. In recent years, new therapeutic strategies such as tumor immunosuppressive therapy have extended patients' lives, and the combination of immune checkpoint inhibitors (ICIs) and VEGF inhibitors is currently positioned as the first-line treatment for advanced HCC.

Immunotherapy has opened a new era of tumor therapy, and immunocheckpoint inhibitors (ICIs), including programmed cell death 1(*PD-1*)/programmed cell death ligand 1(*PD-L1*) inhibitors, have become a breakthrough in tumor therapy. ICIs are typical immunotherapies that activate anti-tumor immunity by inhibiting negative regulatory receptors such as *PD-1* and cytotoxic T lymphocyte antigen 4(*CTLA4*) [11].

At present, immunotherapy for hepatocellular carcinoma has received more and more attention. The occurrence and development of hepatocellular carcinoma is closely related to tumor microenvironment [12]. Tumor microenvironment is a dynamic system composed of tumor cells, complex cytokine environment, extracellular matrix and immune cell subsets [13].

In this study, we constructed and validated an immune-related prognostic model based on the TCGA-LIHC dataset and ICGC-LIRI-JP dataset. In addition, we explored the relationship between the constructed prognostic model and the clinical and pathological features of HCC patients. We analyzed the characteristics of tumor immune microenvironment, including tumor-infiltrating cell composition, immune escape, TP53 mutation rate, and tumor microenvironment. These findings may provide new insights into novel therapeutic targets for hepatocellular carcinoma.

## Materials and methods

### Data preparation and processing

From TCGA (https://portal.gdc.cancer.gov/) download the mRNA expression data and clinical data of LIHC. We obtained the data of 374 tumor specimens and 50 normal specimens. Excluding HCC samples with a survival time of less than 30 days, we finally obtained the data of 342 HCC patients. 342 TCGA tumor samples were randomly divided into two equal parts: training set (Set1) and verification set (set2). The total samples of TCGA were used as another verification set (set3). From ICGC (https://dcc.icgc.org/) download the data of the Japan Institute of liver cancer (ICGC-LIRI-JP), exclude patients with metastatic liver cancer and survival of less than 30 days, and finally 229 patients with HCC were included in the study. These sample data were used as an external validation set (Set4).

From the Gene List module of the Immunology Database and Analysis Portal (ImmPort) database, we downloaded complete gene names directly, totaling 1793 immune-related genes.

### Differential expression analysis and prognostic gene screening

Based on the data of LIHC in TCGA, we analyzed the mRNA expression differences between 374 tumor samples and 50 normal samples. The "Limma" R package was used to screen out differential genes (DEGs) according to adjust *P* < 0.01 and |logFC|> 2. The differentially expressed immune genes (DEIGs) were obtained by the intersection of immune-related genes and DEGs of TCGA. Then DEIGs were obtained by univariate Cox analysis to explore the relationship between overall survival (OS) and gene expression level. When *P* value < 0.05, genes were considered to have significant prognostic potential.

### Copy number variation and functional enrichment analysis

The "RCircos" R package was used to show the mutation locations of these prognostic DEIGs on 23 chromosomes. The gain or loss of these genes were visualized. The "org.hs.eg.db", "Enrichment plot" and "clusterProfiler" packages in R were used to analyze GO and KEGG enrichment of prognostic DEIGs in TCGA to explore potential molecular mechanisms and biological functions [14–16].

### Establishment of immune risk scoring signature (irss) for prognosis

In the TCGA train set (set1), LASSO regression was adopted to process prognostic DEIGs to further identify differentially expressed genes with independent prognostic value. Multivariate cox regression analysis was then used to evaluate whether these genes could be used as independent prognostic predictors and finally determine the genes to construct the model. Next, the following formula was used to calculate the risk score for each patient: 

$$\mathrm{risk}\;\mathrm{score}\;=\;\mathrm{expression}\;\mathrm{for}\;\mathrm{each}\;\mathrm{gene}\;\ast\;\mathrm{coefficient}\;\mathrm{for}\;\mathrm{each}\;\mathrm{gene}$$

The sample was divided into high-risk and low-risk groups based on the median value of the risk score.

### Expression validation of immune-related signature genes

After obtaining informed consent from patients, we collected 20 pairs of HCC tissues and paraneoplastic tissues (from the Second Affiliated Hospital of Nanchang University), then cultured one normal hepatocyte line (7702) and four HCC cell lines (LM3, 97H, HepG2 and 7721), and after extracting RNA from the tissues and cells, q-PCR experiments were performed to verify the signature genes. The primer sequences of the signature genes are shown in Table 1.

**Table 1:** Primer sequences used for RT-qPCR

Gene	Sequence (5’-3’)
*GAPDH*	**F:** GGAGCGAGATCCCTCCAAAAT
	**R:** GCTGTTGTCATACTTCTCATGG
*BIRC5*	**F:** CCACCGCATCTCTACATTCAAG
	**R:** AAGTCTGGCTCGTTCTCAGT
*CCR3*	**F:** CTCCCTCTGCTCGTTATGGC
	**R:** AGCCACATTGTAGGGTGTCC
*GAL*	**F:** AGCGACAAGAATGGCCTCAC
	**R:** CGATGTCTTCTGAGGAGGCTG
*GLP1R*	**F:** GAGCATAGGCTGGGGTGTTC
	**R:** ATGGGCAGCCGGATAATGAG
*IL17B*	**F:** TGTGAACCCCTTCACCATGC
	**R:** GCGATGGTCTCCATGACTGC
*MAPT*	**F:** GCTCATTAGGCAACATCCATCAT
	**R:** CGTGGTCTGTCTTGGCTTTG
*NR0B1*	**F:** GTAAAGAGGCGCTACCAGGC
	**R:** CCTGCGCTTGATTTGTGCTC

Immunohistochemistry (IHC) experiments was used to detect protein expression differences, Relative optical density scores were used to compare the differences between the two groups.

### Validation of the risk score with TCGA and ICGC datasets

According to the established IRSS scoring system, the risk score of each sample was calculated. Set1, set2 and set3 groups were divided into high risk group and low risk group respectively according to the median value of risk score. Then ROC curve and Kaplan–Meier curve were drawn to verify the prediction accuracy of the risk scoring model. Nomogram was used to assess the survival risk of HCC patients in TCGA, including gender, age, TNM stage, IRSS and other clinical information. Calibration curves (1, 2 and 3 years) were drawn to assess the accuracy of Nomogram predictions. To further verify the accuracy of this model, we compared it with published prediction models of HCC.

### Genomic alterations analyses

To determine whether risk score levels were associated with specific genomic traits. Copy number variation (CNV) analysis was performed using the TCGA dataset.

### Association between Microsatellite Instability (MSI) and constructed predictive models

The whole TCGA dataset was further analyzed based on the constructed prognostic model after removing the samples without microsatellite status information. Then, according to the microsatellite status information extracted from the phenotypic data, we first compared whether there was any difference in the expression level of MSI in the high and low risk groups, and then divided the total samples into MSI-high and MSI-low groups through the expression level of MSI in each sample, and compared whether there was a significant difference in OS between the two groups. Thus, the relationship between high and low risk groups and OS can be further determined.

### Analysis of Tumor Mutation Burden (TMB) and immune escape

To further verify the relationship between the constructed model and tumor microenvironment and immunotherapy, correlation boxplot was constructed by Pearson correlation analysis to study the impact of risk score on TMB. TIDE scores were compared between the high and low risk groups to analyze whether there was a difference in the efficacy of immune checkpoint blocking treatment between the high and low risk groups.

### Consensus clustering of prognostic genes

To investigate the functions of the seven selected prognostic DEIGs, we clustered the HCC into different groups with “ConsensusClusterPlus” (50 iterations, resample rate 80% and Pearson correlation). Kaplan–Meier survival curve was used to analyze the OS of each subtype, and the clinical data and gene expression levels between subgroups were compared and analyzed, as well as the immune cell infiltration and immune cell content between subgroups.

### Statistical analysis

The data were analyzed by Excel software, and the visualization of DEGs was completed by "ggplot2", "Cairo" and "ggrepel" R packages. The difference in overall survival was calculated by Kaplan–Meier method, and the significant difference was determined by R. Spearman regression analysis by Cox. ROC curve of R package survival was drawn and visualized to calculate AUC [17]. Somatic mutation and CNV data were downloaded from TCGA database. Copy number changes associated with risk scores were analyzed using GISTIC 2. All data and statistical analysis were based on R software 4.1.2, and *P* < 0.05 was considered statistically significant.

## Results

### Prognostic DEIGs screening

**Fig. 1:**
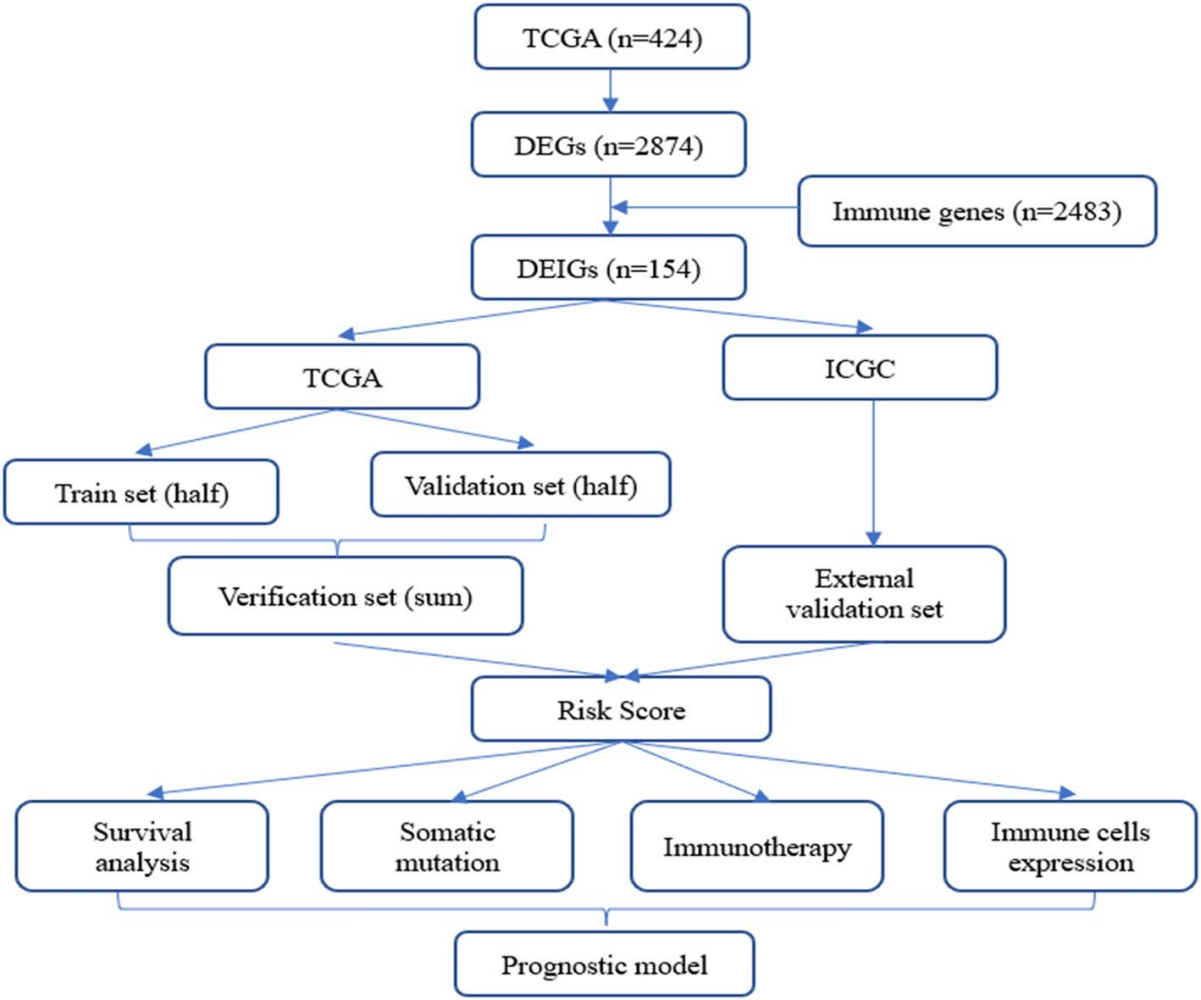
The flow chart of this study

**Fig. 2:**
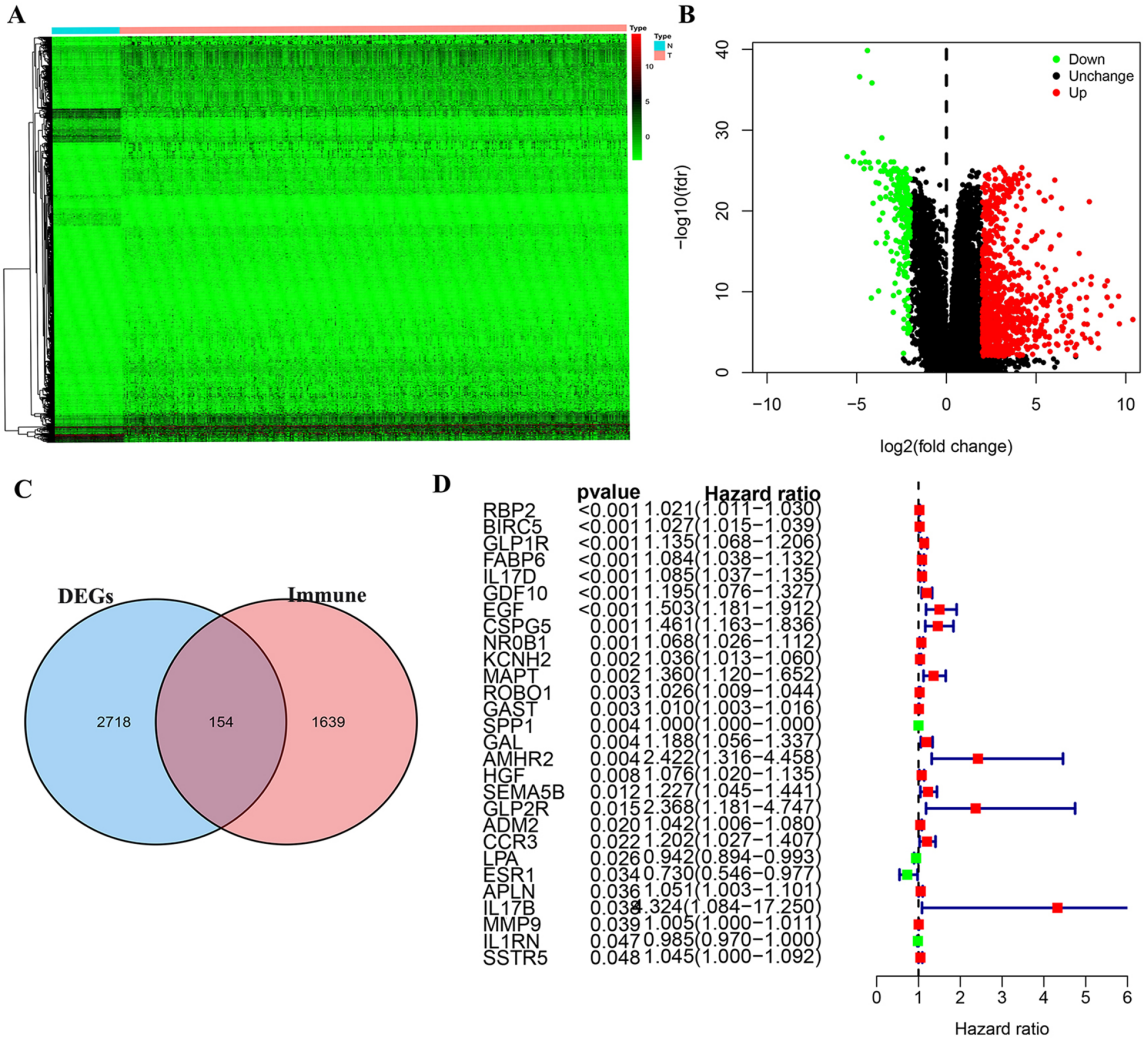
Differentially expressed immune genes (DEIGs) screening and univariate analysis. (**A**) Heatmap for DEGs. (**B**) Volcano plot for DEGs. (**C**) DEIGs scteening. (**D**) Univariate analysis for prognostic genes

The overall process of the study is shown in Fig. 1. We first analyzed the differences between 374 tumor samples and 50 normal samples in TCGA. According to adjust *P* < 0.01 and |logFC|> 2, 2874 differential genes were screened out (Table S1), including 362 down-regulated genes and 2512 up-regulated genes. Visualization using heat and volcano maps (Fig. 2A, B). Then, 154 differential expression immune genes (DEIGs) were obtained through the intersection of immune-related genes (Fig. 2C). Univariate Cox proportional risk analysis was performed on DEIGs, and 28 DEIGs related to prognosis were obtained (Fig. 2D, Table S2).

### Prognostic DEIGs Functional Enrichment and Genetic Alterations

**Fig. 3:**
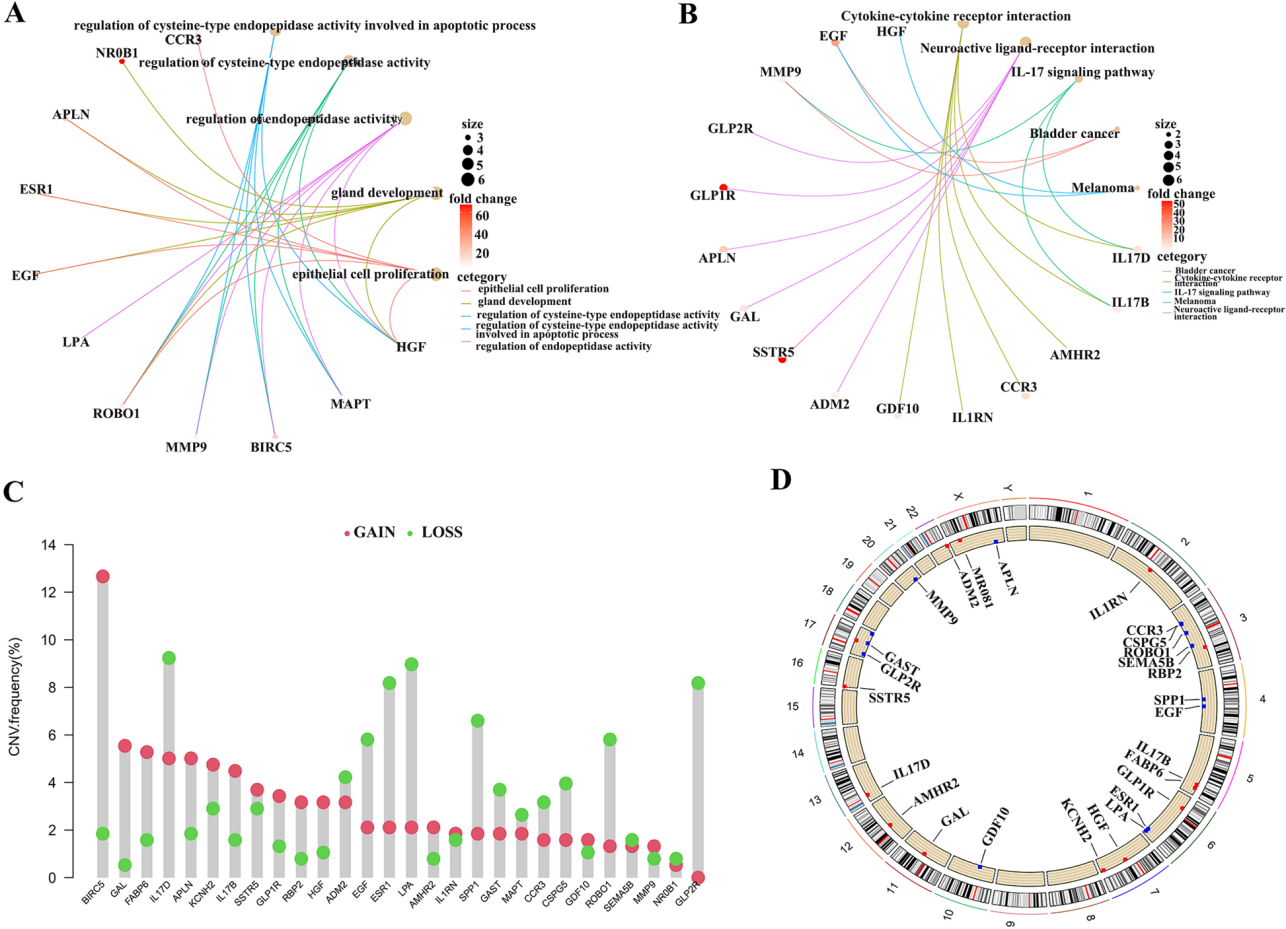
GO, KEGG function enrichment and CNV variation of prognosis-related DEIGs. (**A**) GO function enrichment of 28 prognosis-related DEIGs. (**B**) KEGG pathway enrichment of 28 prognosis-related DEIGs. (**C**) The location of CNV alteration of 28 prognosis-related DEIGs. (**D**) The CNV variation frequency of 28 prognosis-related DEIGs in LIHC

GO and KEGG enrichment analysis was performed on 28 prognostic DEIGs. GO enrichment results showed that prognostic DEIGs were mainly enriched in Epithelial cell proliferation, gland development, regulation of cysteine-type endopeptidase activity, regulation of cysteine-type endopeptidase activity involved in apoptotic process, regulation of endopeptidase activity (Fig. 3A). KEGG results showed that prognostic DEIGs were enriched in Bladder cancer, Cytokine-cytokine receptor interaction, IL-17 signaling pathway, Melanoma, Neuroactive ligand-receptor interaction (Fig. 3B).

We also studied the copy number variation of 28 prognostic genes and summarized the CNV variation frequency of 28 prognostic DEIGs in TCGA-LIHC (Fig. 3C). Visualized the position of CNV variation on chromosomes (Fig. 3D).

### Construction and prognostic value of IRSS

**Fig. 4:**
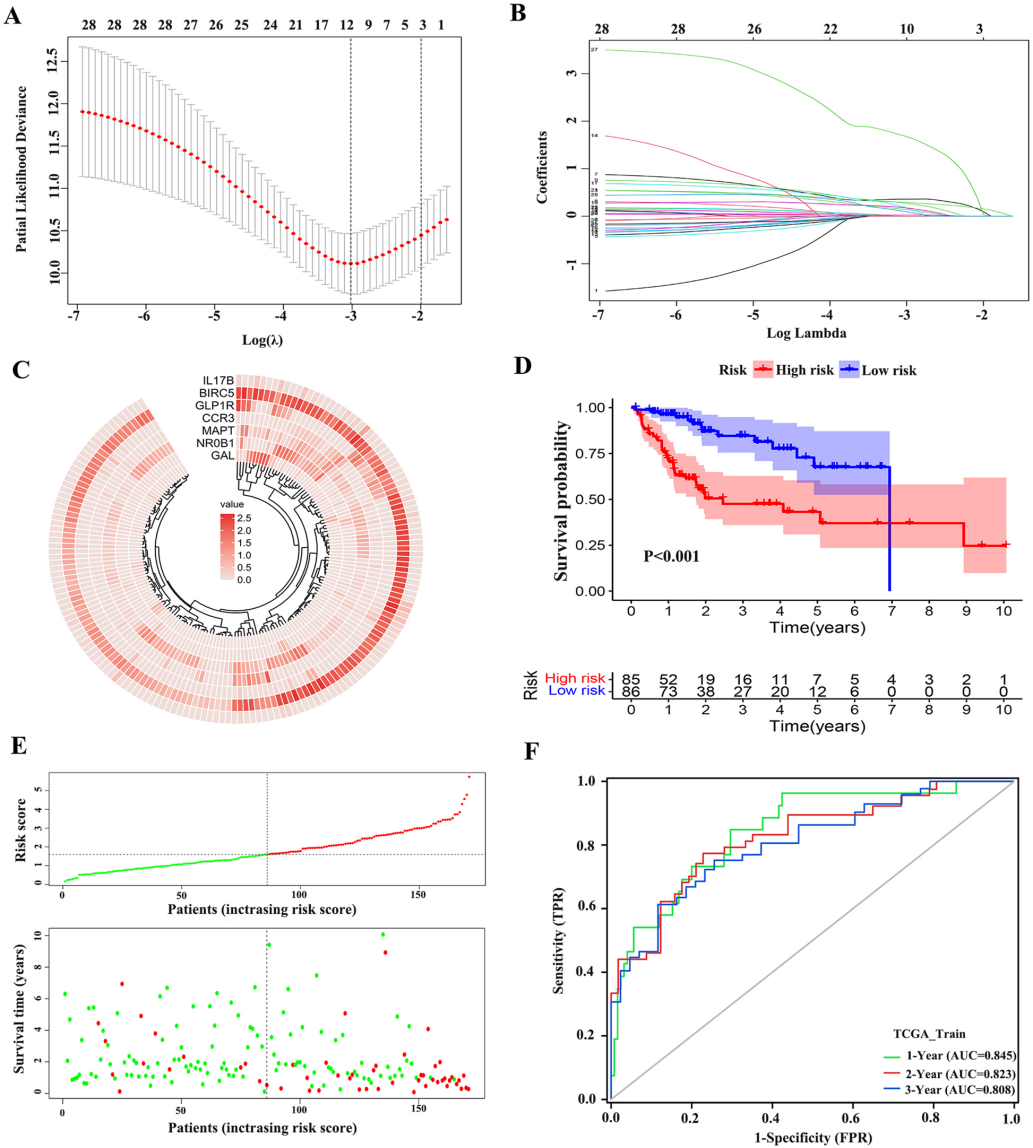
Identification of seven optimal prognosis-related DEIGs. establishment of IRSS. (**A**) Ten-time cross-validation for tuning parameter selection in the lasso model. (**B**) lasso coefficient profiles. (**C**) The expression of 7 genes in TCGA train set. (**D**) Kaplan–Meier survival plots of high-risk and low-risk groups in TCGA train set. (**E**) The risk score and survival status of seven genes in patients. (**F**) Receiver operating characteristic curve (ROC) of risk score in the TCGA train set

In order to calculate the risk score, patients with no survival information and survival time less than 30 days were excluded. Finally, 342 HCC patients were included in the study, and the total sample set (Sum) was randomly divided into the train set and the test set, with 171 patients in each group. Chi-square test was used to determine that there was no statistically significant difference in clinical characteristics among each group (Table 2). LASSO regression analysis was performed on 28 prognostic DEIGs in a training set containing 171 patients, and the model fitted best when the penalty index was 10 (Fig. 4A, B). Then, 7 prognostic genes were obtained through multivariate Cox regression analysis: *GAL*, *NR0B1*, *MAPT*, *CCR3*, *GLP1R*, *BIRC5* and *IL-17B* (Table S3). Combined with the corresponding regression coefficients, the final IRSS is established:

**Table 2:** Chi-square test results of the TCGA training set, the test set and the ICGC cohort

**Characteristics**		**TCGA**		**ICGC** ***n* = 229**	***P*-value**
	**Train (set1)** ***n* = 171**	**Test (set2)** ***n* = 171**	**Sum (set3)** ***n* = 342**		
**Gender**					
Male	125(73.1%)	110(64.3%)	233(68.1%)	168(73.4%)	0.103
Female	46(26.9%)	61(35.7%)	109(31.9%)	61(26.6)	
**Age**					
< = 65	108(63.2%)	109(63.2%)	216(63.2%)	88(38.4%)	0.911
> 65	63(36.8%)	62(36.8%)	126(36.8%)	141(61.6%)	
**Status**					
Alive	113(66.1%)	110(64.3%)	223(65.2%)	189(82.5%)	0.733
Dead	58(33.9%)	61(35.7%)	119(34.8%)	40(17.5%)

$$\mathrm{IRSS}=(GAL\;\exp\;\ast\;0.359)+(NR0B1\;\exp\;\ast\;0.454)+(MAPT\;\exp\;\ast\;0.495)+(CCR3\;\exp\;\ast\;0.641)+(GLP1R\;\exp\;\ast\;0.306)+(BIRC5\;\exp\;\ast\;0.428)+(IL17B\;\exp\;\ast\;2.100)$$

The mRNA expression differences of seven genes in the train group were represented by heat maps (Fig. 4C). The risk score for each patient was calculated based on IRSS, and the sample was divided into high-risk and low-risk groups using the median. In the train set, the probability of OS was lower in the high-risk group than in the low-risk group (*P* < 0.05) (Fig. 4D). As the risk score increased, the survival time decreased and the number of patients in the state of death increased gradually (Fig. 4E). The accuracy of the model in predicting OS of HCC patients was evaluated by ROC curve, and the AUC values at 1, 2 and 3 years were 0.845, 0.823 and 0.808, respectively (Fig. 4F). The mRNA expression differences of 7 prognostic DEIGs in tumor and normal tissues in the TCGA data set (Figure S1).

### Expression validation of Immune-related signature genes

**Fig. 5:**
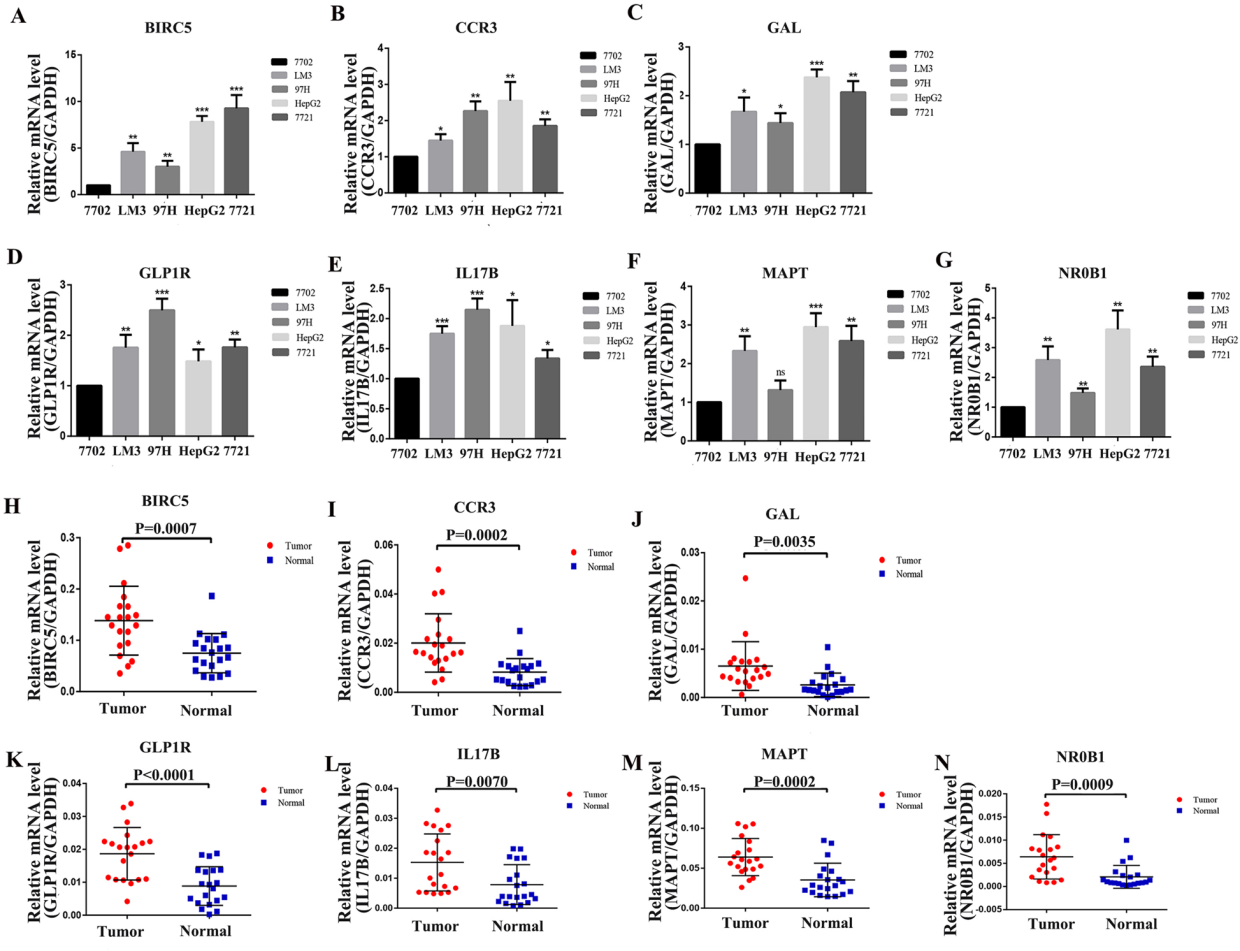
Validation of differential expression of 7 signature genes in cells and tissues by q-PCR. (**A**-**G**) Differential expression of 7 signature genes in 7702, LM3, 97H, HepG2 and 7721 cells. The results showed that the expression of *BIRC5, CCR3, GAL, GLP1R, IL17B, MAPT* and *NR0B1* in HCC cells was significantly higher than that in normal liver cells. (**H**-**N**) Differential expression of 7 signature genes in HCC tissue and paraneoplastic tissue, the expression of *BIRC5, CCR3, GAL, GLP1R, IL17B, MAPT* and *NR0B1* were significantly higher in HCC tissues

**Fig. 6:**
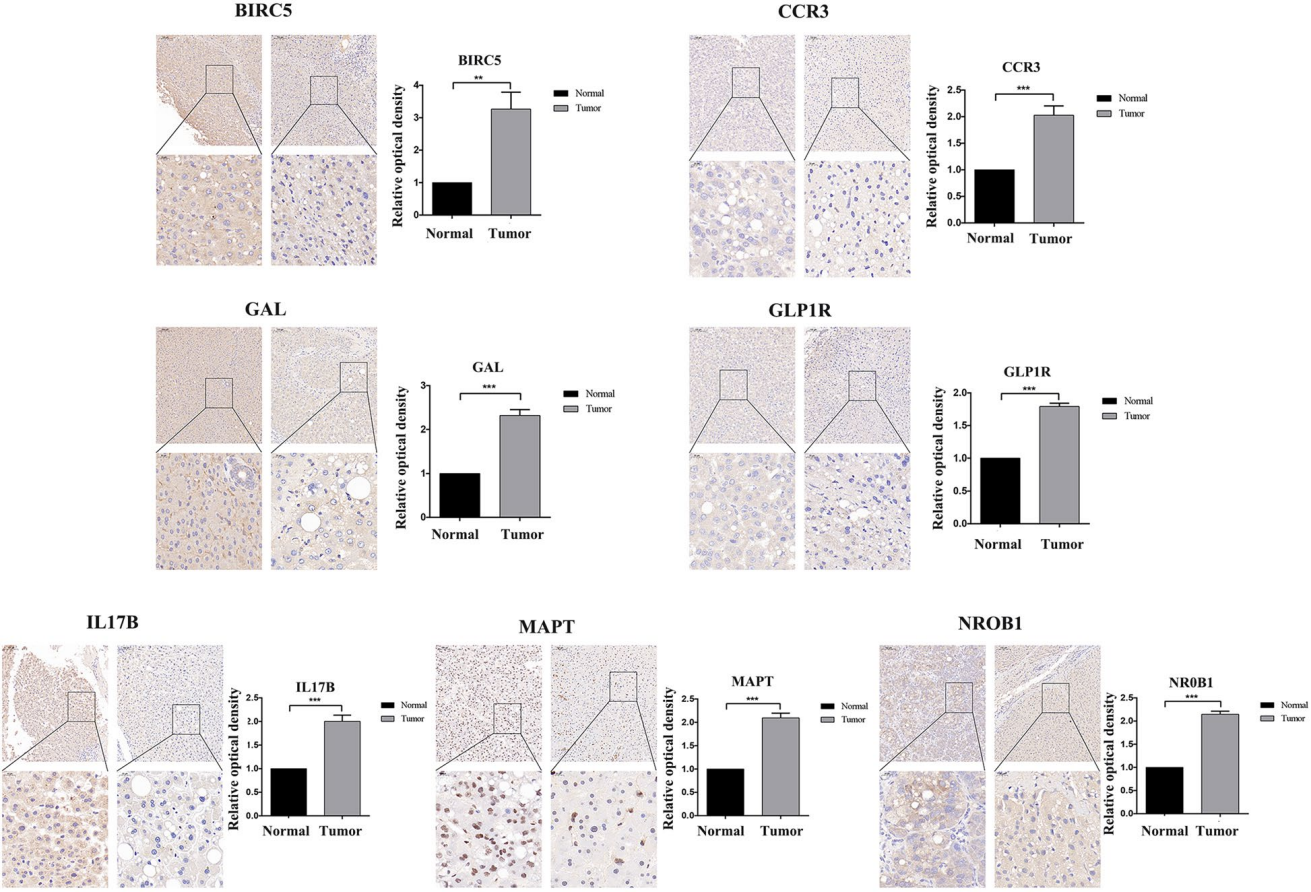
Staining images of seven signature genes in HCC tissues and paraneoplastic tissues. Relative optical density scores were used to compare the differences between the two groups

The expression of the signature genes in cells (normal liver cells vs HCC cells) and tissues (HCC tissues vs paraneoplastic tissues) were verified by q-PCR assay (Fig. 5A-N). and we showed representative images of 7 genes, and then compared the expression differences between HCC tissues and paraneoplastic tissues using relative optical density scores (Fig. 6).

### Validation of The Risk Score with TCGA and ICGC Datasets

**Fig. 7:**
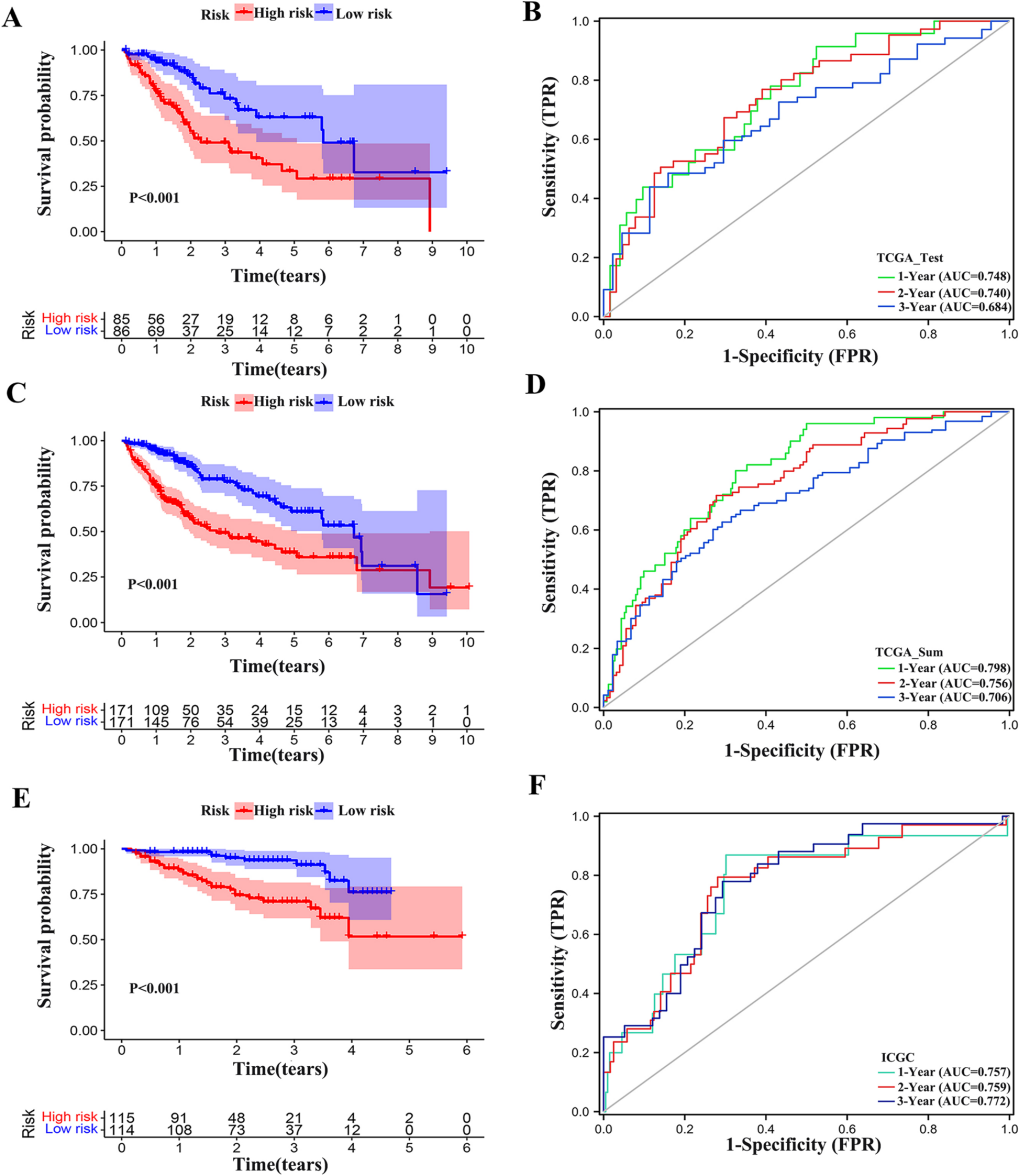
Risk score validation. (**A**) Kaplan–Meier survival plots in TCGA test set. (**B**) ROC of risk score in the TCGA test set. (**C**) Kaplan–Meier survival plots in TCGA sum set. (**D**) ROC of risk score in the TCGA sum set. (**E**) Kaplan–Meier survival plots in ICGC validation set. **(F)** ROC of risk score in the ICGC validation set

In the test set, according to the risk score calculated by IRSS, the samples were divided into high and low risk groups using the median value. The analysis found that there was a significant difference in survival probability between the high and low risk groups (*P* < 0.05), and the high-risk group had a lower survival rate (Fig. 7A). The AUC values assessed by ROC curve at 1, 2 and 3 years were 0.748, 0.740 and 0.684, respectively (Fig. 7B). The mRNA expression differences of prognostic genes in the test set were represented by heat maps (Figure S2A).

In TCGA sum set, the total sample is also divided into high and low risk groups according to IRSS calculated risk score. Analysis showed that there was a significant difference in overall survival between the two groups (*P* < 0.05), and the survival rate was lower in the high-risk group (Fig. 7C). The AUC values of 1, 2 and 3 years were 0.798, 0.756 and 0.706, respectively (Fig. 7D). The mRNA expression differences of prognostic genes in TCGA sets were represented by heat maps (Figure S2B).

**Fig. 8:**
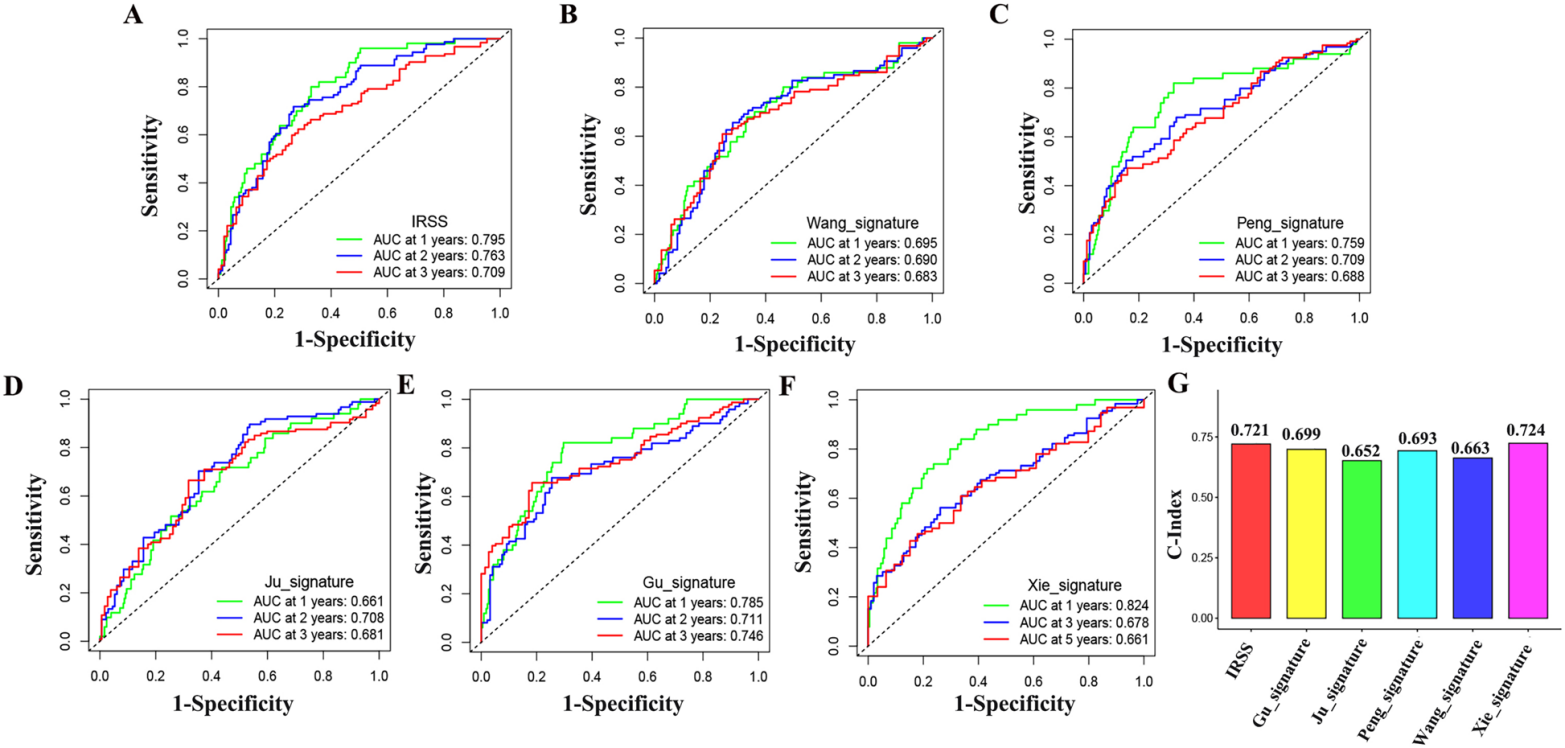
Compared with four prediction models. (**A**) The ROC curve of our IRSS. (**B**) The ROC curve of Wang_signature. (**C**) The ROC curve of Peng_signature. (**D**) The ROC curve of Ju_signature. (**E**) The ROC curve of Gu_signature. (F) The ROC curve of Xie_signature; (**F**) C-index of six prediction models

According to IRSS, ICGC data were divided into high risk group and low risk group, and the survival rate difference between the two groups was also statistically significant (*P* < 0.05) (Fig. 7E). The AUC values of 1, 2 and 3 years were 0.757, 0.759 and 0.772, respectively (Fig. 7F). After multiple validation, we find that the model has high robustness and accuracy. The mRNA expression differences of prognostic genes in the ICGC dataset were represented by heat maps (Figure S2C). Meanwhile, in order to further verify the reliability of the model, we compared it with four published prediction models of HCC [18–22]. It turns out that our model has a high score (Fig. 8A-G).

### Independent prognostic analysis

**Fig. 9:**
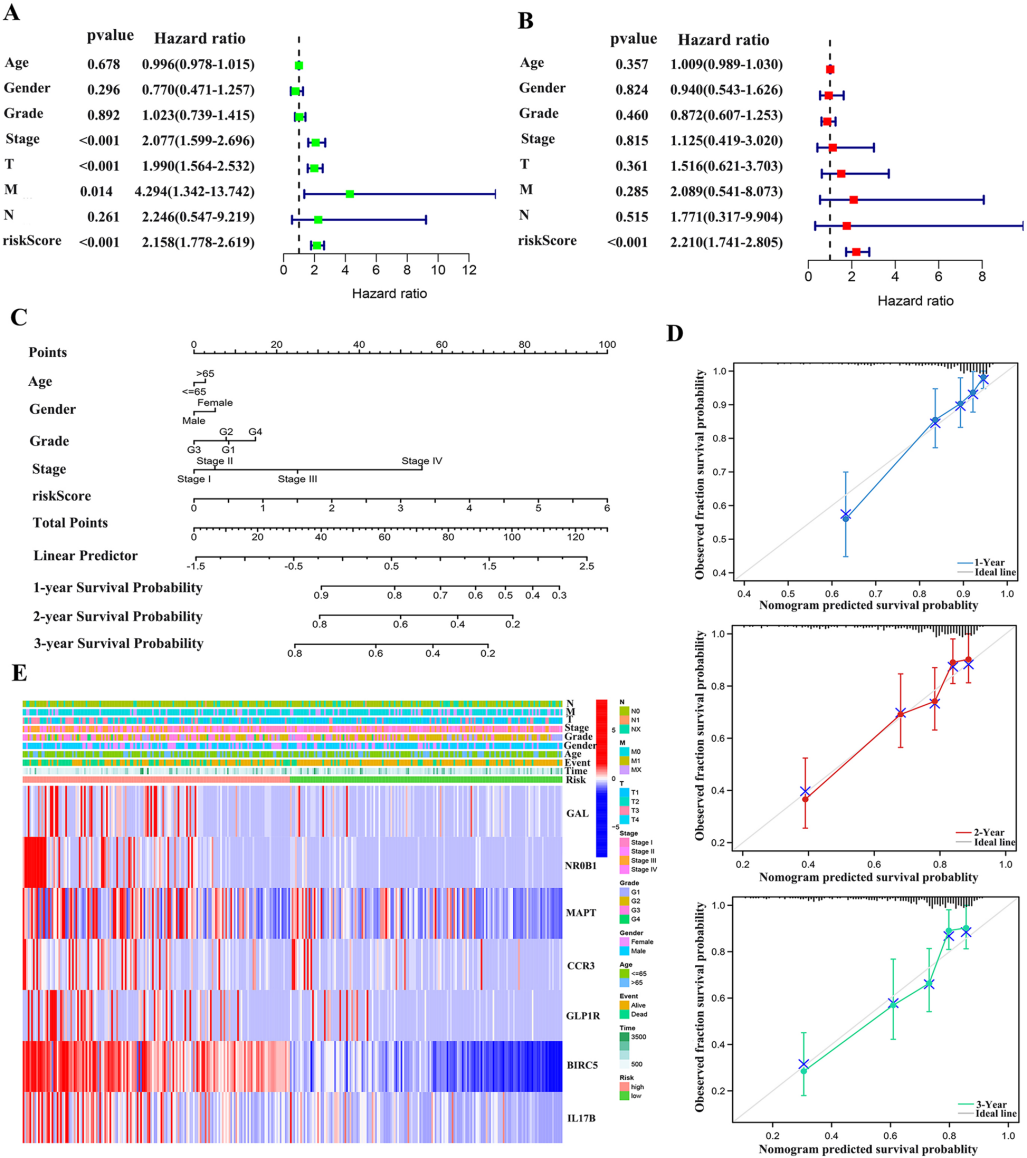
Selection of the independent prognostic factors. (**A**) Forest plot of univariate cox regression analysis in TCGA. (**B**) Forest plot of multivariate cox regression analysis in TCGA. (**C**) 1-,2- and 3-year nomogram for predicting OS of LIHC. (**D**) Decision curve analysis for the evaluation of the net benefits of IRSS and nomogram at 1, 2 and 3 year. (**E**) Heatmap of seven genes and clinical factors

To verify the reliability of the risk factors, independent prognostic analyses were performed. In univariate Cox analysis, risk score, Stage and T Stage were significantly correlated with OS (*P* < 0.05) (Fig. 9A). In multivariate Cox analysis, only risk score was confirmed as an independent predictor of OS (Fig. 9B). The above results again demonstrate the stability of the IRSS established by us. In order to further evaluate individual patients, Nomograms were used to simplify the statistical prediction model to comprehensively predict the prognosis of HCC patients by calculating the scores of clinical data and risk scores (Fig. 9C, D).

In addition, we demonstrated the correlation between clinical features and risk in heat map (Fig. 9E). Boxplot was used to show the clinical indicators and risk scores and the differences between the high and low risk groups. There were no significant differences in age, gender, N stage and M stage in the high and low risk groups (*P* > 0.05). Stage, Grade and T Stage were later with the increase of risk score (Figure S3).

### Genomic alterations analyses

**Fig. 10:**
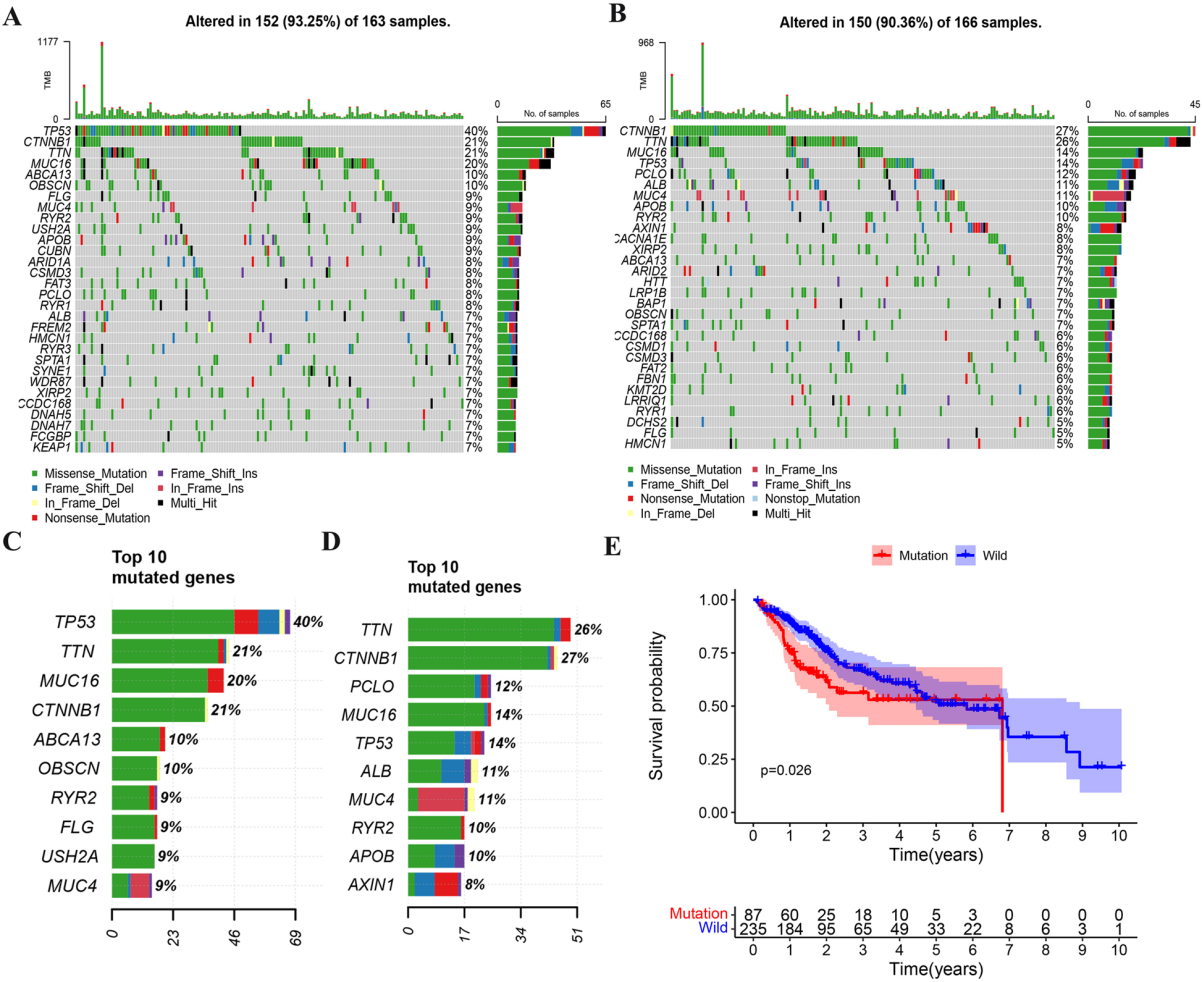
Analysis of somatic mutation profiles based on risk score levels. (**A**) CNV profile in the high riskScore group. (**B**) CNV profile in the low riskScore group. (**C**) Top ten mutated genes in the high riskScore group. (**D**) Top ten mutated genes in the low riskScore group. (**E**) The association between the TP53 status and patients’ OS in LIHC

To determine whether risk score levels were associated with specific genomic traits, CNV and somatic mutation analyses were performed using the TCGA data set. According to the risk score levels, *TP53*(40%), *CTNNB1*(21%), *TTN* (21%) and *MUC16* (20%) had the highest mutation frequency in the high-risk group (Fig. 10A, B). In the low-risk group, *CTNNB1*(27%), *TTN* (26%), *MUC16* and *TP53* (14%) were more frequent, and the mutation rate of *TP53* was significantly higher in the high-risk group (Fig. 10C, D). *TP53* is a well-known tumor suppressor gene, which is usually associated with poor prognosis [23]. Therefore, we also conducted survival analysis on TP53 mutation data, and the results showed that the survival rate of *TP53* mutation group was significantly lower (*P* < 0.05) (Fig. 10E).

### Correlation analysis between tumor microenvironment and stem cells

**Fig. 11:**
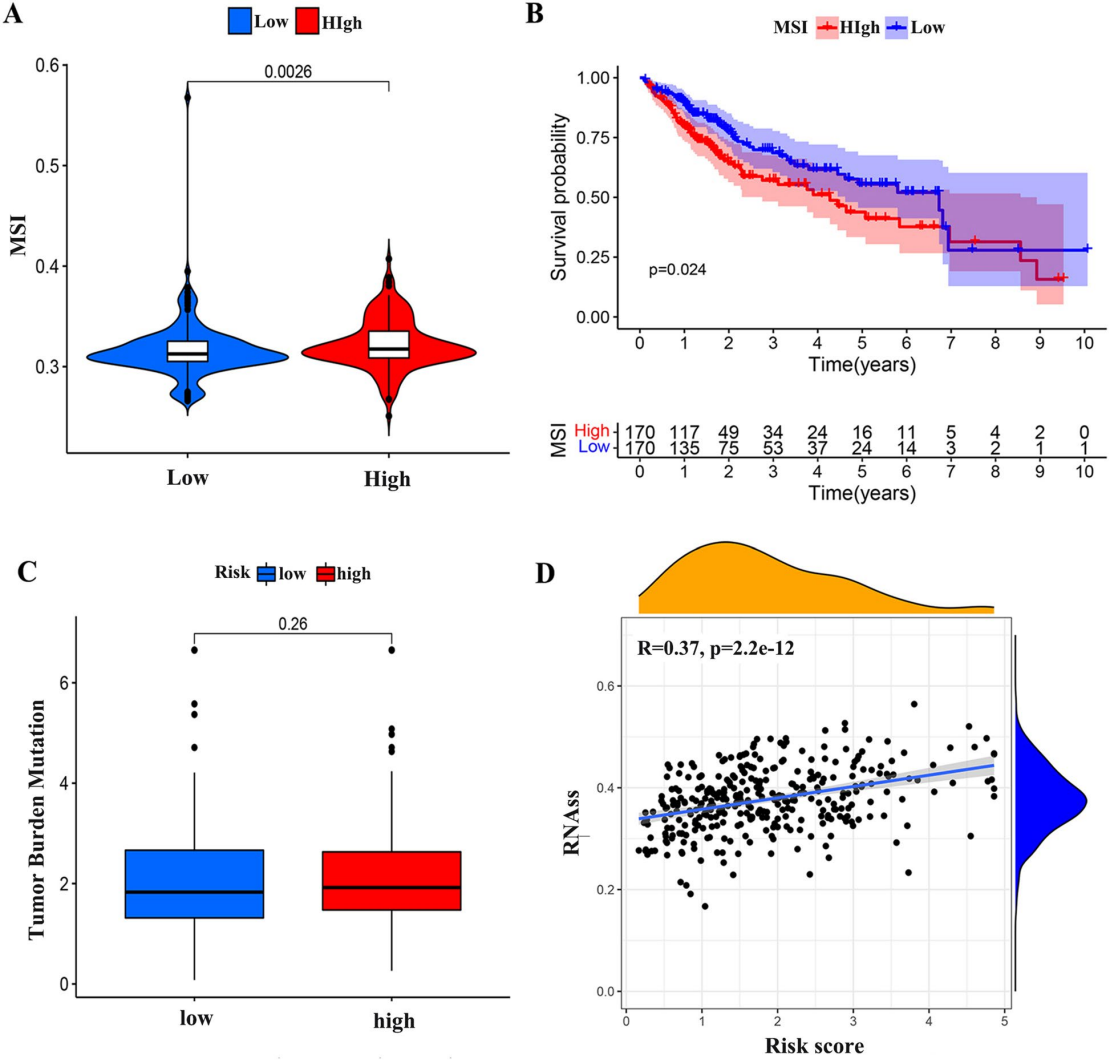
Exploring the immune microenvironment and stem cells in HCC. (**A**) The difference of MSI between the high-risk and low-risk group. (**B**) Relationship between MSI expression level and survival of patients. (**C**) The difference of TMB between the high-risk and low-risk group. (**D**) The correlation between risk score and tumor stem cells

Analysis of tumor microenvironment suggested that the expression level of tumor microsatellite instability (MSI) was significantly different in the high-risk group, and higher in the high-risk group (Fig. 11A). In order to explore the relationship between MSI and survival of HCC, we divided the samples into the high MSI expression group and the low MSI expression group according to the expression level of MSI. Survival analysis of the two groups showed that the high MSI expression group had a lower survival rate (*P* < 0.05) (Fig. 11B), which further confirmed the low survival rate of the high-risk group. There was no significant difference in tumor mutation burden (TMB) between high and low risk groups (Fig. 11C).

In recent years, tumor stem cells have been considered as the root cause of tumor occurrence, metastasis and recurrence. We analyzed the association between risk score and tumor stem cells and found that there was a significant correlation between the two. The higher the risk score, the higher the score of tumor stem cells (*P* < 0.05) (Fig. 11D). It can be inferred that the higher the risk score, the lower the degree of tumor differentiation.

### Immunotherapy analysis

**Fig. 12:**
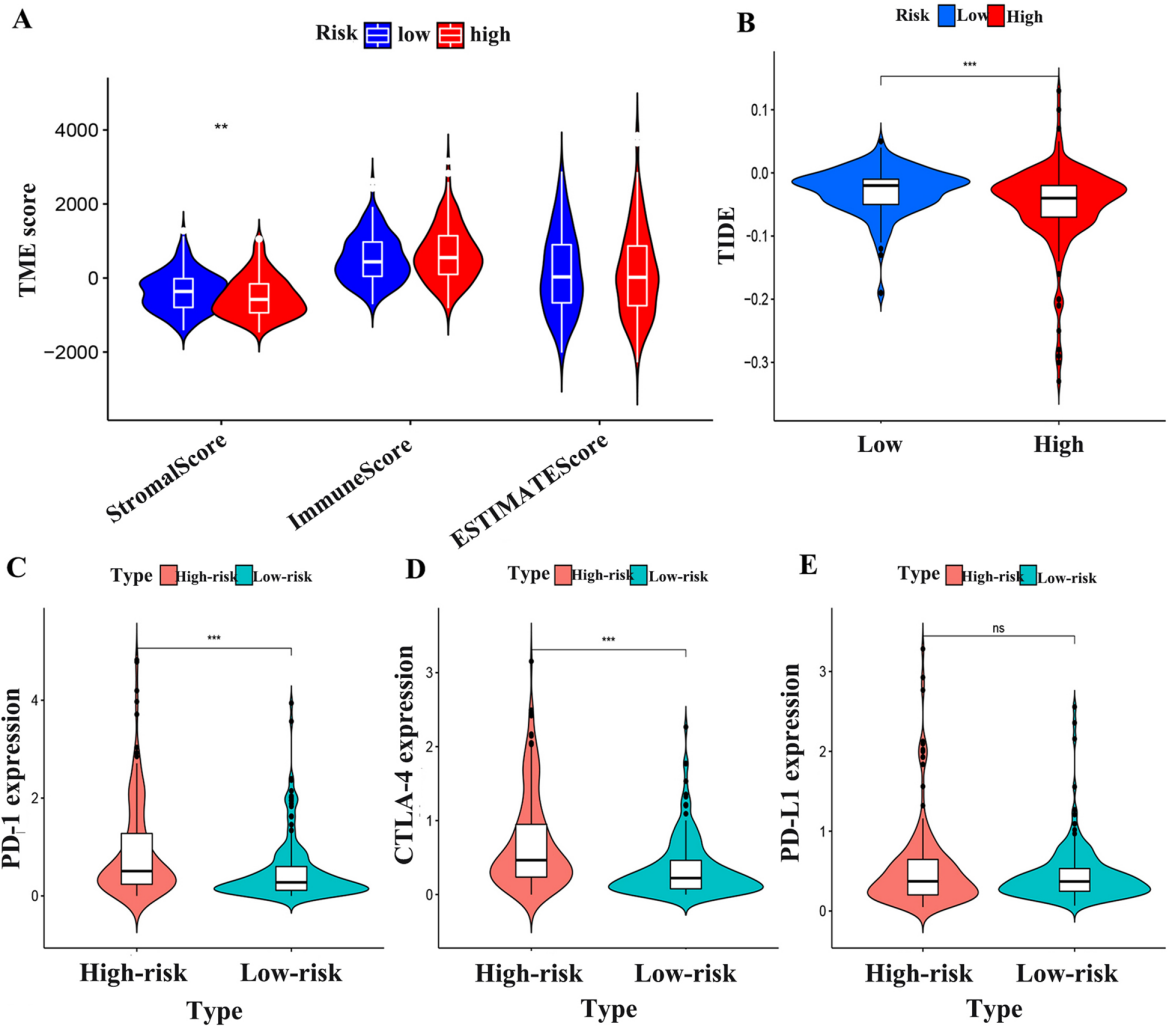
Exploring the immunotherapy in HCC patients. (**A**) Stromal and Immune cells scores in LIHC. (**B**) Comparison of the scores of TIDE between the high-risk and low-risk group. (**C**) The difference of PD-1 between the high-risk and low-risk group. (**D**) The difference of CTLA-4 between the high-risk and low-risk group. (**E**) The difference of PD-L1 between the high-risk and low-risk group

The infiltration of immune cells and stromal cells in HCC tissues was analyzed according to the risk score groups, and the results showed that there was significant difference in stromal cell score between high-risk and low-risk groups (*P* < 0.05), but no significant difference in immune cells (*P* > 0.05) (Fig. 12A). Comparison of TIDE scores showed that there were fewer dysfunction and immune rejection T cells in the high-risk group for HCC (Fig. 12B). In order to explore the relationship between the efficacy of immune checkpoint inhibitors and risk score, the expression of *PD-1* and *CTLA-4* in the high-risk group was significantly higher than that in the low-risk group (*P* < 0.05) by analyzing the expression differences of immune checkpoint between the high-risk group and the low-risk group (Fig. 12C, D). There was no significant difference in *PD-L1* between the two groups (*P* > 0.05) (Fig. 12E).

### Consensus clustering of seven prognostic genes

**Fig. 13:**
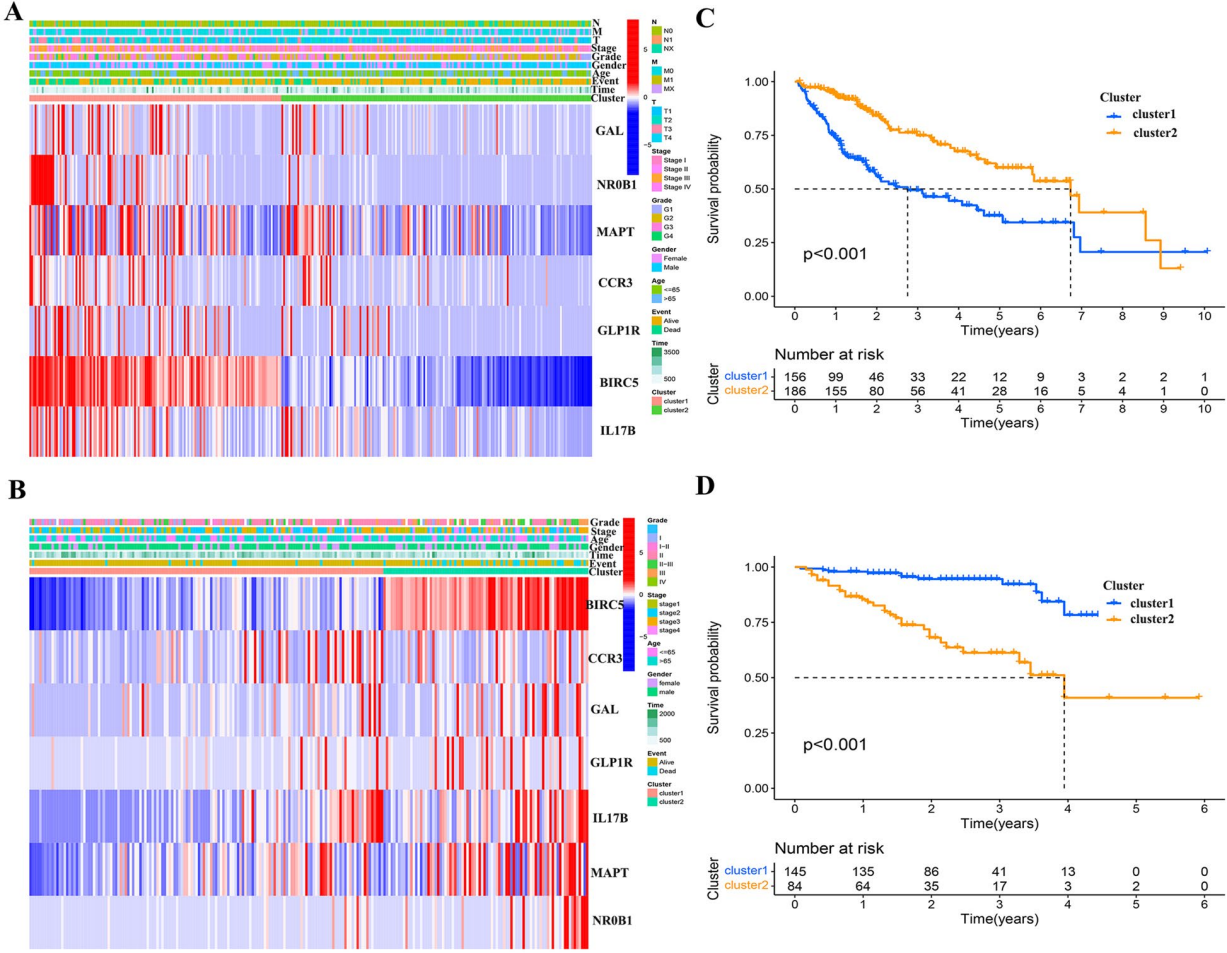
Consensus clustering and overall survival in TCGA and ICGC. (**A**) Heatmap showing sample clusters with distinct clinical outcomes, clinical features and pathological features in TCGA. (**B**) Kaplan–Meier overall survival curves using TCGA data. (**C**) Heatmap showing sample clusters with distinct clinical outcomes, clinical features and pathological features in ICGC. (**D**) Kaplan–Meier overall survival curves using ICGC data

Consensus clustering of the seven prognostic DEIGs identified two clusters of HCC in the TCGA and CGGA datasets with distinct clinical outcomes, clinical features and pathological features (Fig. 13A, B). According to expression similarity, k = 2 was selected with clustering stability rising from k = 2 to 10 in the TCGA and ICGC datasets. A contingency table showed consistency between clustered groups and risk groups in both TCGA and ICGC datasets (Figure S4). In the TCGA and ICGC datasets, the survival difference between the two clusters was significant (Fig. 13C, D). Between groups, PCA distribution was clearly separated in the TCGA and CGGA datasets (Figure S5A, B). The relationship between each subtype and risk score and prognosis is shown in Figure S5C, D.

### Differential analysis of immune cell composition

**Fig. 14:**
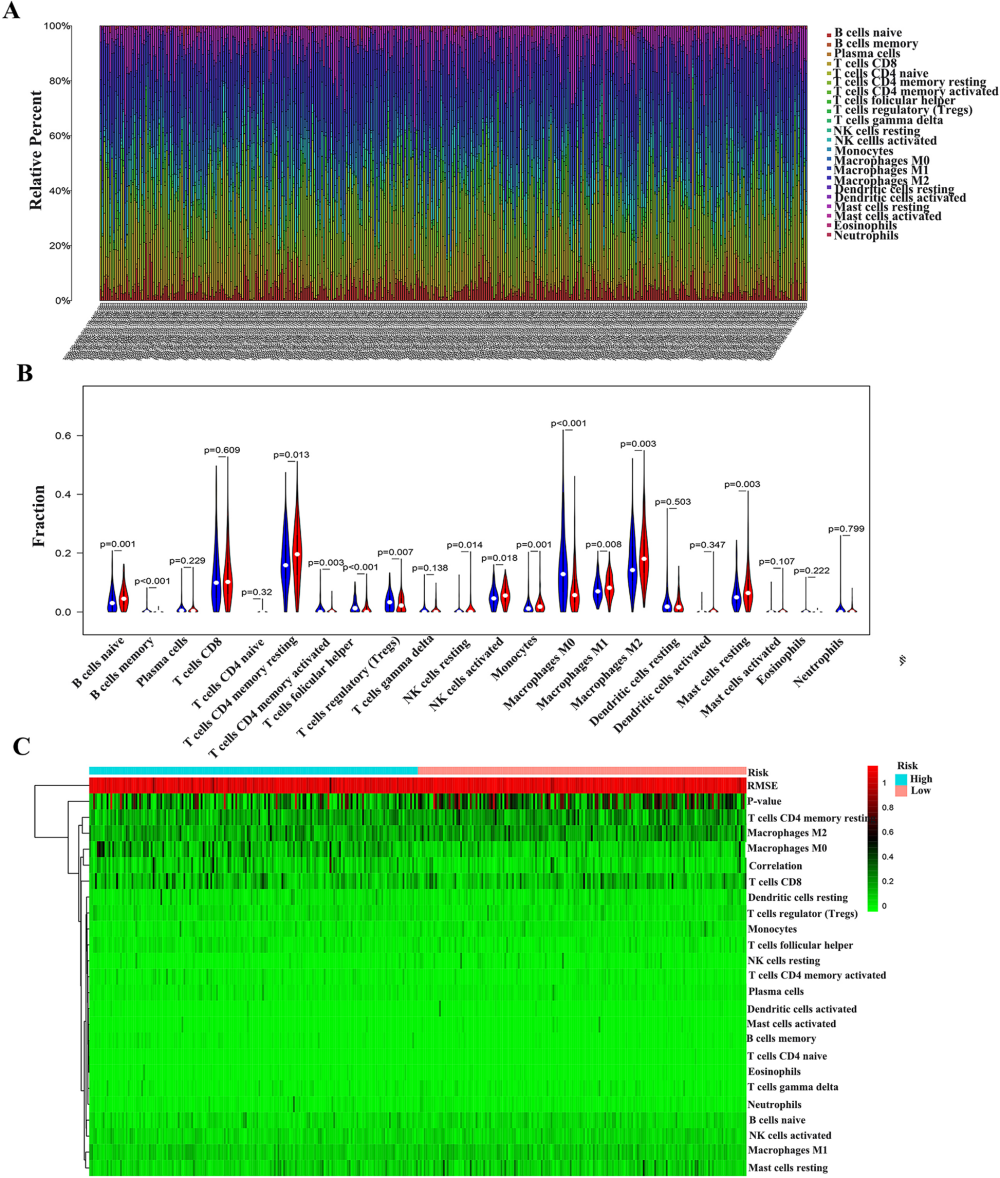
The relationship between risk score and immune cell composition in the TCGA-LIHC data. (**A**) The proportion of composition by 22 immune cell types in tumor samples is shown in the stacked histogram. (**B**) Immune cell composition in the high-risk and low-risk group, as analyzed using the CIBERSORT algorithm. (**C**) The relationship between immune cell composition and risk score

Firstly, the content of immune cells in each sample in the TCGA dataset was analyzed and shown in a histogram (Fig. 14A). By analyzing these seven immune genes and immune function, we found that risk score was correlated with immune cell regulation, and in the high-risk group, B cells naive, T cells CD4 memory resting, NK cells activated, monocyte, Macrophages M1, Macrophages M2, Mast cells resting significantly increased (*P* < 0.05), B cells memory, T cells CD4 memory activated, T cells follicular helper, T cells regulatory (Tregs), M0 of Macrophages was significantly decreased (*P* < 0.05) (Fig. 14B). The correlation between immune cells and 7 prognostic genes is shown in Figure S6. In addition, we used heat maps to show differences in immune cell content between the high and low risk groups (Fig. 14C).

## Discussion

By studying and analyzing the differential immunity genes related to prognosis in HCC, we aim to further explore the targets that can effectively improve prognosis and therapeutic effect of hepatocellular carcinoma. The treatment of HCC is a global challenge, and its molecular pathogenesis varies with different genotoxic lesions and diseases. Although our understanding of the pathophysiology and drivers of disease has improved, this knowledge has not yet been translated into clinical practice [24]. About 25% of HCC patients have operable mutations, but the incidence of most mutations is less than 10%, which makes the study more complicated [25].

Currently, systemic therapies including immune checkpoint inhibitors (ICIs), tyrosine kinase inhibitors (TKI), and monoclonal antibodies challenge the use of conventional therapies for HCC. Tumors often upregulate immune checkpoints to avoid detection and killing by the host immune system. Activation of checkpoint cascades, such as those controlled by *PD-1* or *CTLA-4*, leads to tumor-specific T cell inactivation and immune evasion [26, 27]. Treatment with anti-*PD-1*, anti-*PD-L1* or anti-*CTLA-4* revitalizes T cells and allows the adaptive immune system to target tumor cells [28, 29]. Inhibitors of *PD1, PDL1,* and *CTLA-4* are pillars of clinical practice or systemic therapies under development for HCC. Data from the CheckMate-040 Phase I/II trial, presented at the American Society of Clinical Oncology Annual Meeting 2019, The combination of Opdivo (PD-1 Antibody) and Yervoy (CTLA-4 Antibody) yielded an objective response rate of 31% and a response time of 17.5 months [30]. This result suggests that immunotherapy has a potential and surprising effect on HCC.

Tumor microenvironment (TME) of HCC refers to a complex and spatially structured mixture of liver parenchymal resident cells, tumor cells, immune cells and tumor-associated fibroblasts [31]. These populations interact dynamically through intercellular contact and the release or recognition of cytokines and other soluble factors. This complex cellular interaction has a significant impact on tumor immune evasion. TMB is often used as a proxy for the number of neoantigens because the probability of recognizing neoantigen-specific T lymphocytes is associated with TMB [32]. The frequency of these genetic changes for each type varies greatly between individual tumors and between tumor types [33]. TMB can be used to predict Immune checkpoint blockade (ICB) efficacy and has become a useful biomarker for multiple cancer types to identify patients who will benefit from immunotherapy [34].

*GAL, NR0B1, MAPT, CCR3, GLP1R, BIRC5* and *IL17B* were the Prognostic DEIGs included in the prognostic signature. which can preliminarily predict the prognosis and immunotherapy effect of HCC patients by analyzing their relationship with tumor microenvironment, immune invasion and immunotherapy. It is helpful for the systemic treatment of HCC. Anti-*GAL* antibodies have been confirmed to play a role in the immunotherapy of pancreatic cancer [35]. *NR0B1* has also been confirmed to be related to the occurrence and development of a variety of tumors, for example, the transformed phenotype of Ewing's sarcoma requires sustained NR0B1 expression [36]. Knockdown of *NR0B1* can reduce the tumorigenic and anti-apoptotic potential of lung adenocarcinoma [37]. *MAPT* is a microtubule-related protein tau, which can inhibit the function of taxanes, and its high expression reduces the sensitivity to taxanes, which is of great significance in breast cancer research [38]. Studies have also confirmed that *MAPT* is often methylated, and hypermethylation is associated with poor prognosis in patients with stage II colorectal cancer [39]. Both *CCR3* and *IL17B* have been found to be related to the role of tumor microenvironment in regulating tumor growth and metastasis, and may be new immunotherapy targets [40, 41]. Previous studies have shown that glucagon-like peptide-1 receptor (*GLP1R*) is essential for the regulation of glucose homeostasis, and in recent years, it has been found to be related to the occurrence and development of tumors [42, 43]. *BIRC5* has been confirmed to be related to the occurrence of a variety of tumors, and its role in the progression of liver cancer has also been confirmed by studies [44]. However, there are few studies on its immunity to liver cancer, which is worth exploring.

In addition, by exploring tumor immune microenvironment and gene mutations, our study found that mutation rates of MSI and TP53 may also be independent prognostic indicators. The higher the risk score, the lower the survival rate. However, patients also had a lower immune escape frequency fraction, suggesting that the high-risk group may have a better effect on immunotherapy. The expression levels of *PD-1* and *CTLA-4* were higher in the high-risk group. Therefore, the treatment effect of anti-*PD-1* and anti-*CTLA-4* is better, which is expected to improve the prognosis of patients. Up to now, there are many prognostic models for HCC, but due to the complexity of HCC, no one model has been considered as the gold standard. Our study focused on the prognosis and immunoassay of hepatocellular carcinoma, combined with the current hot spot of immunotherapy, aiming to find effective immunotherapy targets for hepatocellular carcinoma. The disadvantages of this study are as follows: The clinical data in TCGA and ICGC databases did not specify in detail whether patients had received chemotherapy or radiotherapy, which may have certain influence on patient survival data and immunoassay results.

## Conclusion

In summary, a robust immune-related prognosis model was constructed and tumor microenvironment and immune function were analyzed, providing potential targets for immunotherapy of HCC.

## Supplementary Information

**Additional file 1:** **Table S1.** Difference analysis between normal and tumor groups of LIHC.

**Additional file 2:** **Table S2.** Univariate cox regression for DEIGs.

**Additional file 3:** **Table S3.** The multivariate Cox regression analysis.

**Additional file 4:** **Figure S1.** The expression of seven IRSS genes between normal and tumor.

**Additional file 5:** **Figure S2.** Heat map results of seven IRSS genes expression levels.The expression of seven IRSS genes in the TCGA test set.The expression of seven IRSS genes in the TCGA sum set.The expression of seven IRSS genes in the ICGC validation set.

**Additional file 6:** **Figure S3.** The differences in risk scores across clinical features.Age.Gender.Grade.Stage.T stage.N stage.M stage.

**Additional file 7:** **Figure S4.** Identification of consensus clusters by prognostic genes.Consensus cluster matrix for k=2 in TCGA dataset.Relative change in area under CDF curve for k=2 to 10 in TCGA dataset.Consensus clustering cumulative distribution functionfor k=2 to 10 in TCGA dataset.Consensus cluster matrix for k=2 in ICGC dataset.Relative change in area under CDF curve for k=2 to 10 in ICGC dataset.Consensus clustering cumulative distribution functionfor k=2 to 10 in ICGC dataset.Contingency table showing the consistency between clustered groups and risk groups in TCGA.Contingency table showing the consistency between clustered groups and risk groups in ICGC.

**Additional file 8:** **Figure S5.** Clustering analyses in TCGA and ICGC.PCA results for two groups of patients in TCGA.ggalluvial of two clusters in TCGA displayed the correlation between clusters, risk, and survival status.PCA results for two groups of patients in ICGC.ggalluvial of two clusters in ICGC displayed the correlation between clusters, risk, and survival status.

**Additional file 9:** **Figure S6.** Correlation between immune cells and seven genes in TCGA. MAPT and NROB1 have no Significant correlation wirh immune cells in TCGA.

## Data Availability

The dataset supporting the conclusions of this article is included within the article and its supplementary file. TCGA-LIHC is available at https://portal.gdc.cancer.gov/; ICGC-LIRI-JP is available at https://dcc.icgc.org/releases/current/Projects/LINC-JP.
