# Supplementary figures and images for: Signature construction and molecular subtype identification based on immune-related genes for better prediction of prognosis in hepatocellular carcinoma
Source: BMC Med Genomics. 2023 Jun 14;16:130. doi: 10.1186/s12920-023-01558-z (PMC10265900; doi:10.1186/s12920-023-01558-z)

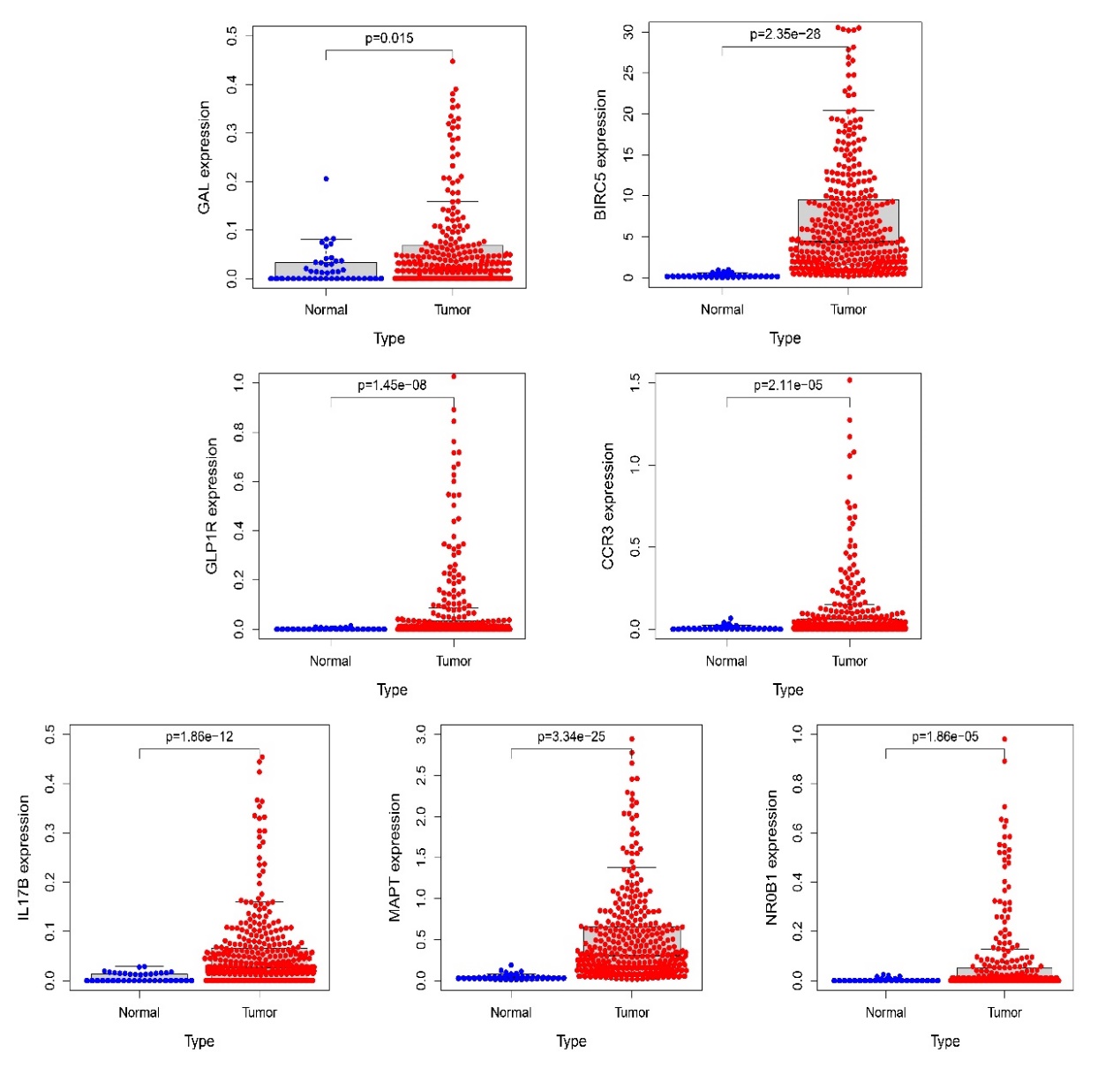


**Figure S1 |** The expression of seven IRSS genes between normal and tumor.

Supplement: Supplementary file 4 — Additional file 4: Figure S1. The expression of seven IRSS genes between normal and tumor. [file 12920_2023_1558_MOESM4_ESM.docx]
