# Supplementary material for: Signature construction and molecular subtype identification based on immune-related genes for better prediction of prognosis in hepatocellular carcinoma
Source: BMC Med Genomics. 2023 Jun 14;16:130. doi: 10.1186/s12920-023-01558-z (PMC10265900; doi:10.1186/s12920-023-01558-z)
Supplement: Supplementary file 5 — Additional file 5: Figure S2. Heat map results of seven IRSS genes expression levels.The expression of seven IRSS genes in the TCGA test set.The expression of seven IRSS genes in the TCGA sum set.The expression of seven IRSS genes in the ICGC validation set. [file 12920_2023_1558_MOESM5_ESM.docx]

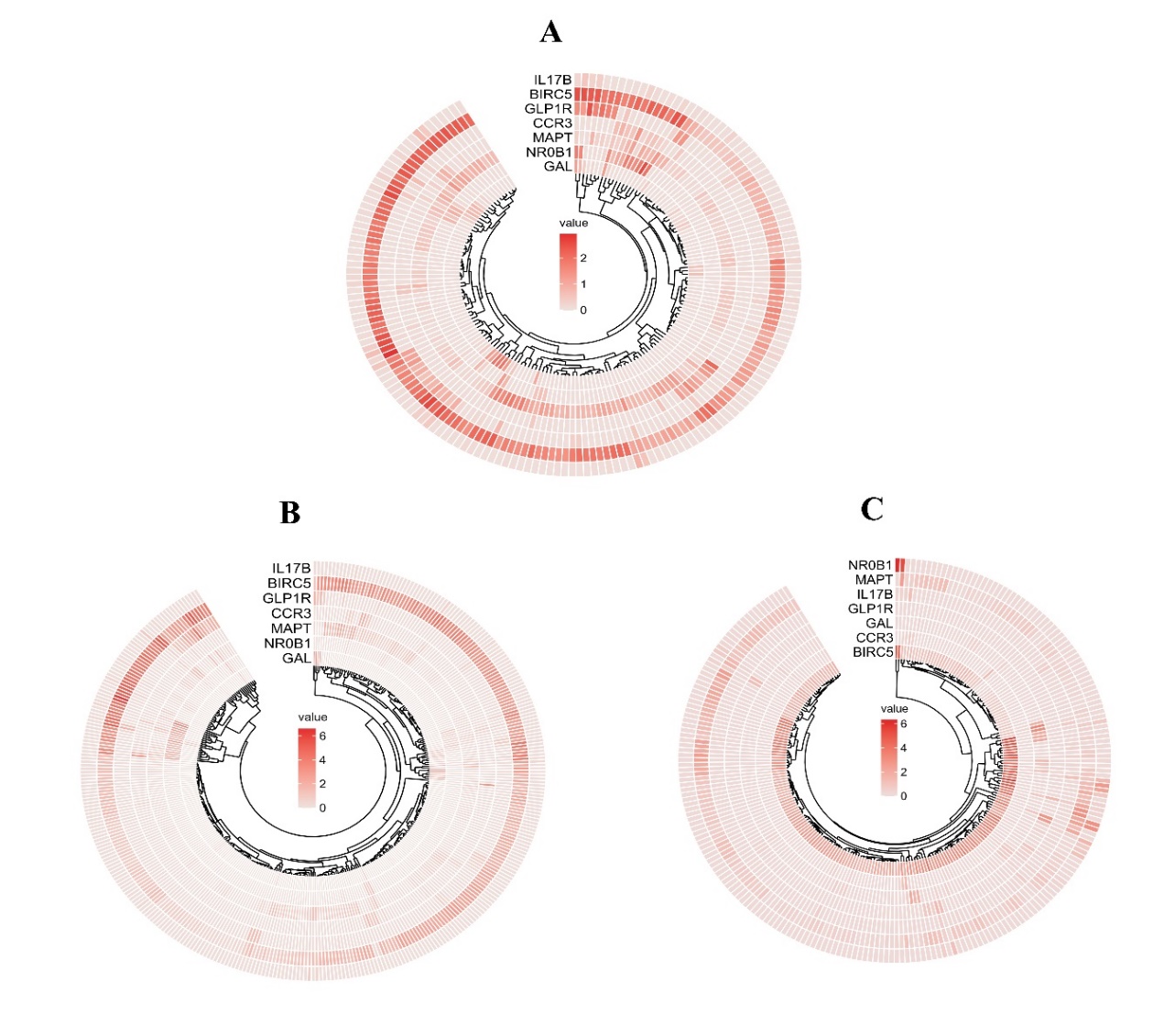


**Figure S2 |** Heat map results of seven IRSS genes expression levels.**(A)** The expression of seven IRSS genes in the TCGA test set. **(B)** The expression of seven IRSS genes in the TCGA sum set. **(C)** The expression of seven IRSS genes in the ICGC validation set.
