# Supplementary material for: Signature construction and molecular subtype identification based on immune-related genes for better prediction of prognosis in hepatocellular carcinoma
Source: BMC Med Genomics. 2023 Jun 14;16:130. doi: 10.1186/s12920-023-01558-z (PMC10265900; doi:10.1186/s12920-023-01558-z)
Supplement: Supplementary file 6 — Additional file 6: Figure S3. The differences in risk scores across clinical features.Age.Gender.Grade.Stage.T stage.N stage.M stage. [file 12920_2023_1558_MOESM6_ESM.docx]

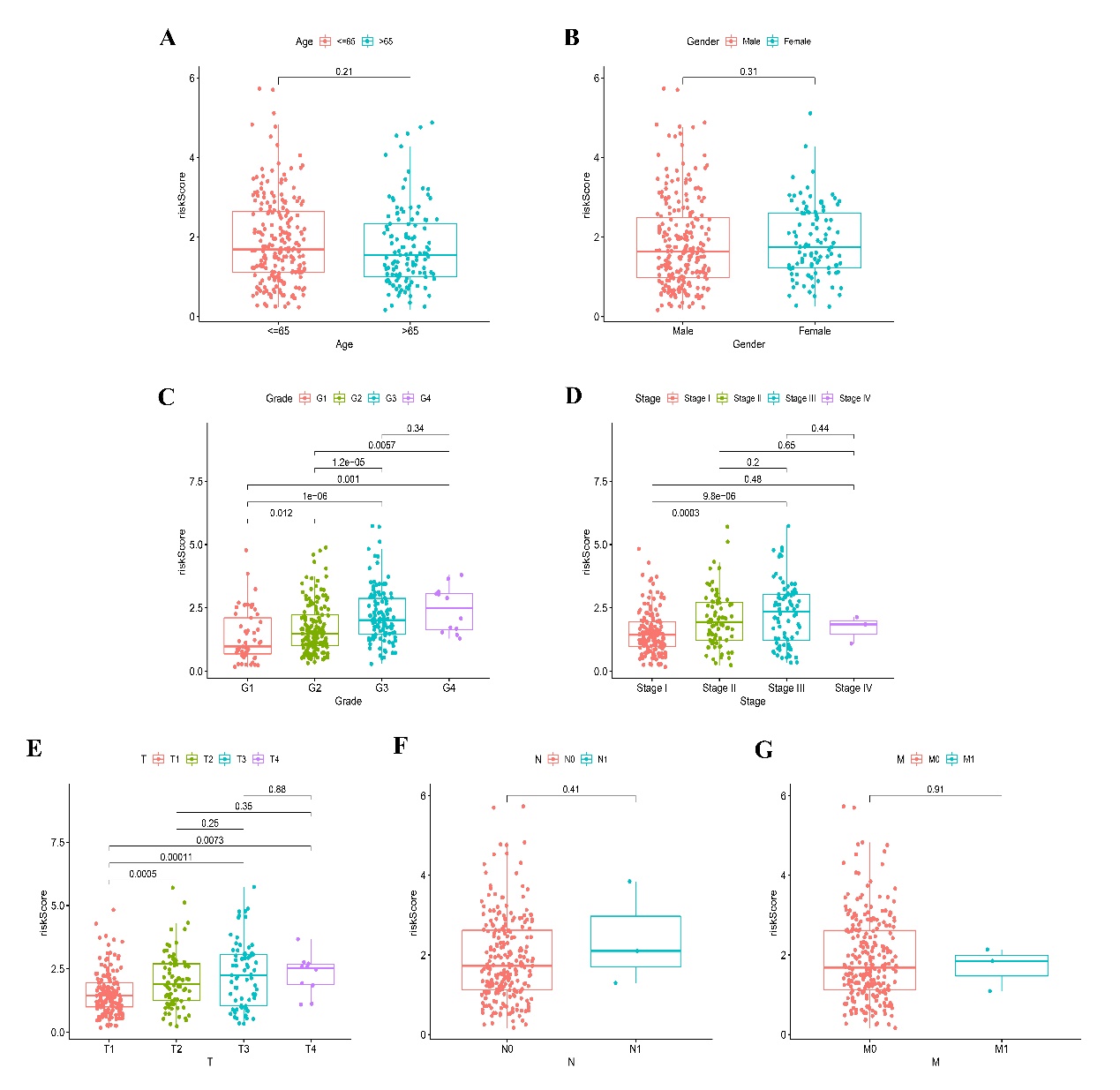


**Figure S3 |** The differences in risk scores across clinical features. **(A)** Age. **(B)** Gender. **(C)** Grade. **(D)** Stage. **(E)** T stage. **(F)** N stage. **(G)** M stage.
