# Supplementary material for: Signature construction and molecular subtype identification based on immune-related genes for better prediction of prognosis in hepatocellular carcinoma
Source: BMC Med Genomics. 2023 Jun 14;16:130. doi: 10.1186/s12920-023-01558-z (PMC10265900; doi:10.1186/s12920-023-01558-z)
Supplement: Supplementary file 7 — Additional file 7: Figure S4. Identification of consensus clusters by prognostic genes.Consensus cluster matrix for k=2 in TCGA dataset.Relative change in area under CDF curve for k=2 to 10 in TCGA dataset.Consensus clustering cumulative distribution functionfor k=2 to 10 in TCGA dataset.Consensus cluster matrix for k=2 in ICGC dataset.Relative change in area under CDF curve for k=2 to 10 in ICGC dataset.Consensus clustering cumulative distribution functionfor k=2 to 10 in ICGC dataset.Contingency table showing the consistency between clustered groups and risk groups in TCGA.Contingency table showing the consistency between clustered groups and risk groups in ICGC. [file 12920_2023_1558_MOESM7_ESM.docx]

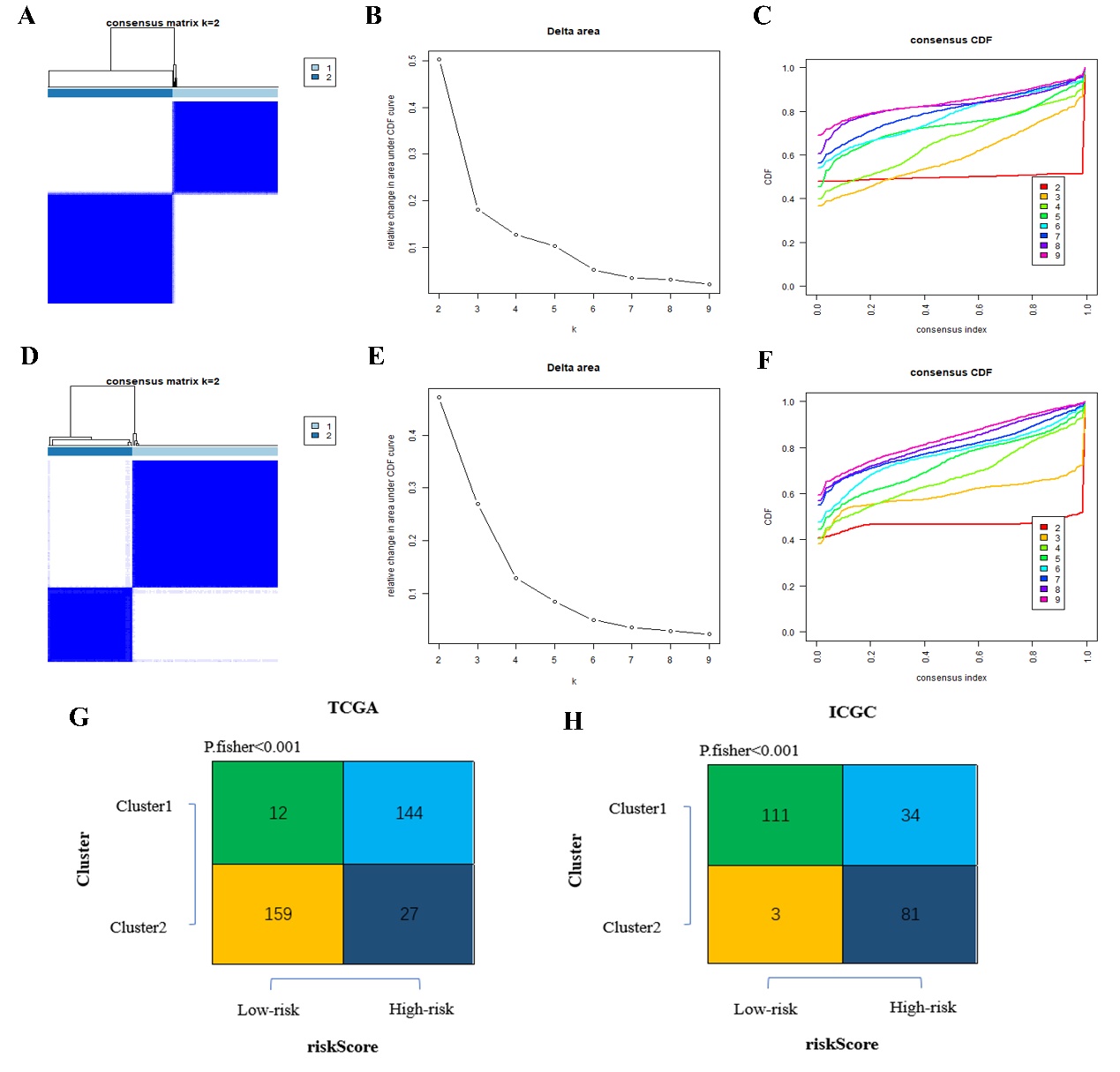


**Figure S4 |** Identification of consensus clusters by prognostic genes. **(A)** Consensus cluster matrix for k=2 in TCGA dataset. **(B)** Relative change in area under CDF curve for k=2 to 10 in TCGA dataset. **(C)** Consensus clustering cumulative distribution function (CDF) for k=2 to 10 in TCGA dataset. **(D)** Consensus cluster matrix for k=2 in ICGC dataset. **(E)** Relative change in area under CDF curve for k=2 to 10 in ICGC dataset. **(F)** Consensus clustering cumulative distribution function (CDF) for k=2 to 10 in ICGC dataset. **(G)** Contingency table showing the consistency between clustered groups and risk groups in TCGA. **(H)** Contingency table showing the consistency between clustered groups and risk groups in ICGC.
