# Supplementary material for: Signature construction and molecular subtype identification based on immune-related genes for better prediction of prognosis in hepatocellular carcinoma
Source: BMC Med Genomics. 2023 Jun 14;16:130. doi: 10.1186/s12920-023-01558-z (PMC10265900; doi:10.1186/s12920-023-01558-z)
Supplement: Supplementary file 8 — Additional file 8: Figure S5. Clustering analyses in TCGA and ICGC.PCA results for two groups of patients in TCGA.ggalluvial of two clusters in TCGA displayed the correlation between clusters, risk, and survival status.PCA results for two groups of patients in ICGC.ggalluvial of two clusters in ICGC displayed the correlation between clusters, risk, and survival status. [file 12920_2023_1558_MOESM8_ESM.docx]

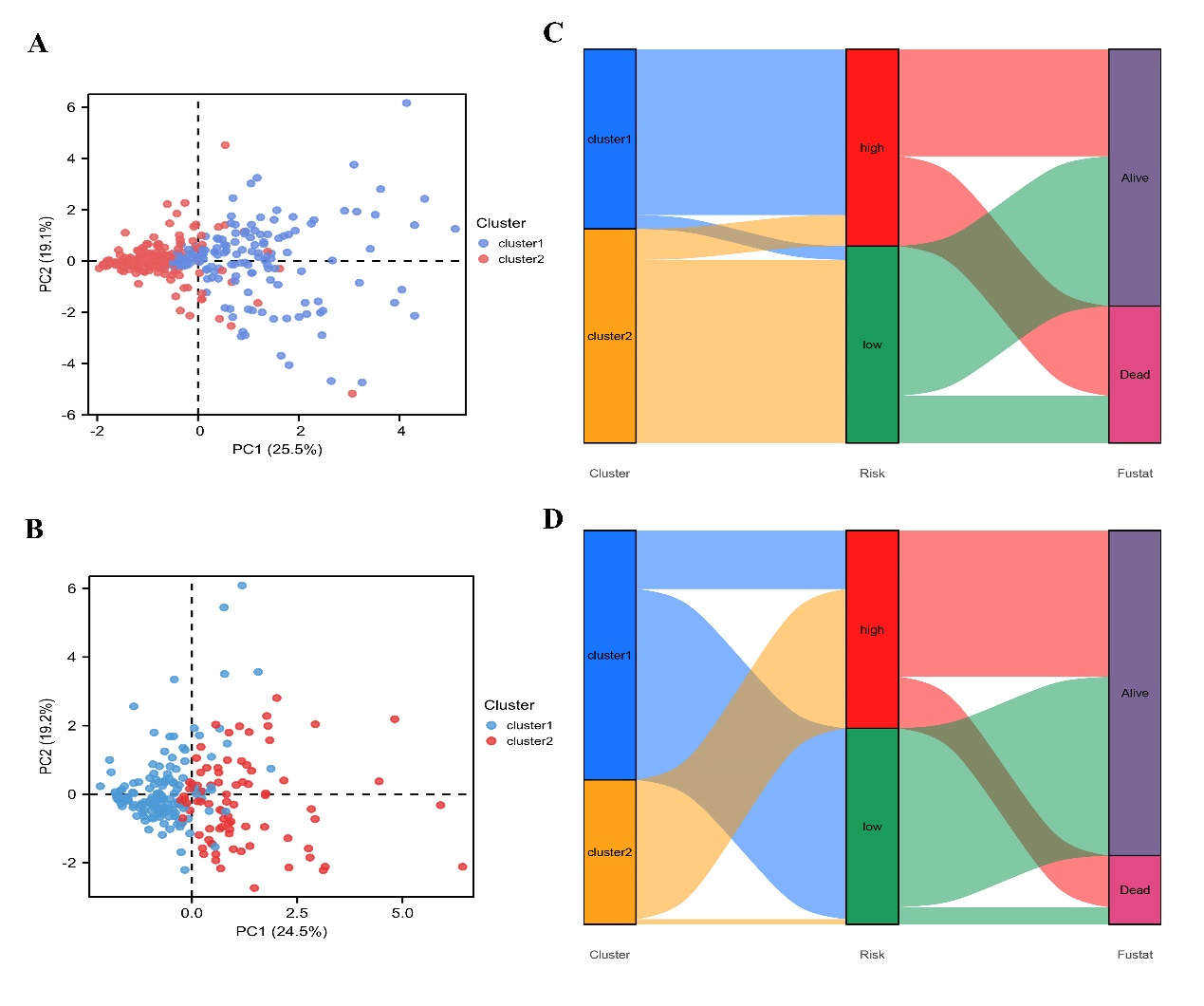


**Figure S5 |** Clustering analyses in TCGA and ICGC. **(A)** PCA results for two groups of patients in TCGA. **(B)** ggalluvial of two clusters in TCGA displayed the correlation between clusters, risk, and survival status. **(C)** PCA results for two groups of patients in ICGC. **(D)** ggalluvial of two clusters in ICGC displayed the correlation between clusters, risk, and survival status.
