# Supplementary material for: Signature construction and molecular subtype identification based on immune-related genes for better prediction of prognosis in hepatocellular carcinoma
Source: BMC Med Genomics. 2023 Jun 14;16:130. doi: 10.1186/s12920-023-01558-z (PMC10265900; doi:10.1186/s12920-023-01558-z)
Supplement: Supplementary file 9 — Additional file 9: Figure S6. Correlation between immune cells and seven genes in TCGA. MAPT and NROB1 have no Significant correlation wirh immune cells in TCGA. [file 12920_2023_1558_MOESM9_ESM.docx]

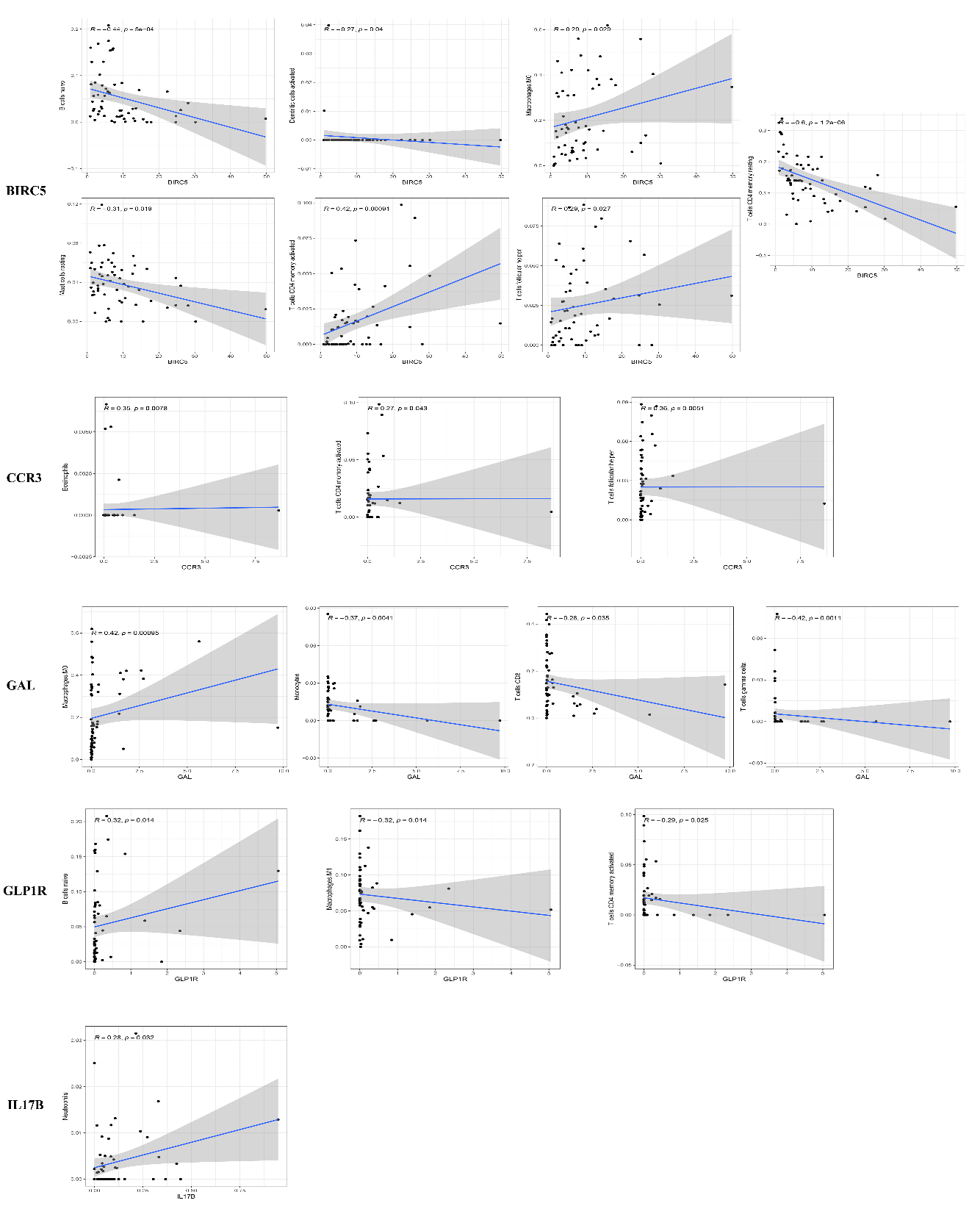


**Figure S6 |** Correlation between immune cells and seven genes in TCGA. MAPT and NROB1 have no Significant correlation wirh immune cells in TCGA.
